# A spectral dimension reduction technique that improves pattern detection in multivariate spatial data

**DOI:** 10.1093/bioinformatics/btag052

**Published:** 2026-01-31

**Authors:** David Köhler, Niklas Kleinenkuhnen, Kiarash Rastegar, Till Baar, Chrysa Nikopoulou, Vangelis Kondylis, Vlada Milchevskaya, Matthias Schmid, Peter Tessarz, Achim Tresch

**Affiliations:** University of Bonn, University Hospital Bonn, Institute for Medical Biometry, Informatics, and Epidemiology, Bonn 53127, Germany; Institute of Medical Statistics and Computational Biology, Faculty of Medicine, University of Cologne, Cologne 50931, Germany; Institute of Medical Statistics and Computational Biology, Faculty of Medicine, University of Cologne, Cologne 50931, Germany; Max Planck Research Group ‘Chromatin and Ageing’, Max Planck Institute for Biology of Ageing, Cologne 50931, Germany; Institute of Medical Statistics and Computational Biology, Faculty of Medicine, University of Cologne, Cologne 50931, Germany; Institute of Medical Statistics and Computational Biology, Faculty of Medicine, University of Cologne, Cologne 50931, Germany; Max Planck Research Group ‘Chromatin and Ageing’, Max Planck Institute for Biology of Ageing, Cologne 50931, Germany; Institute for Pathology, University Hospital Cologne, Cologne 50937, Germany; Institute of Medical Statistics and Computational Biology, Faculty of Medicine, University of Cologne, Cologne 50931, Germany; University of Bonn, University Hospital Bonn, Institute for Medical Biometry, Informatics, and Epidemiology, Bonn 53127, Germany; Max Planck Research Group ‘Chromatin and Ageing’, Max Planck Institute for Biology of Ageing, Cologne 50931, Germany; Cologne Excellence Cluster on Cellular Stress Responses in Aging-Associated Diseases (CECAD), University of Cologne, Cologne 50931, Germany; Institute of Medical Statistics and Computational Biology, Faculty of Medicine, University of Cologne, Cologne 50931, Germany; Cologne Excellence Cluster on Cellular Stress Responses in Aging-Associated Diseases (CECAD), University of Cologne, Cologne 50931, Germany; Center for Data and Simulation Science, University of Cologne, Cologne 50931, Germany

## Abstract

**Motivation:**

We introduce a statistical approach for pattern recognition in multivariate spatial transcriptomics data.

**Results:**

Our algorithm constructs a projection of the data onto a low-dimensional feature space which is optimal in maximizing Moran’s I, a measure of spatial dependency. This projection mitigates non-spatial variation and outperforms principal components analysis for pre-processing. Patterns of spatially variable genes are well represented in this feature space, and their projection can be shown to be a denoising operation. Our framework does not require any parameter tuning, and it furthermore gives rise to a calibrated, powerful test of spatial gene expression.

**Availability and implementation:**

The algorithm is implemented in the open source software R and is available at https://github.com/IMSBCompBio/SpaCo.

## 1 Introduction

Technological advances have made spatially resolved transcriptomics widely applicable in the life sciences, providing near-cellular resolution of tissue heterogeneity and organization. Neighbouring cells in a tissue are typically of similar types, leading to a dependence of observations on proximity. This has spurred research on strategies for handling the rich data from spatial omics, in particular for the detection of spatially variable genes (SVGs) (Chang *et al.* 2024, Yuan *et al.* 2024) and the construction of non-redundant features for dimension reduction (Shang and Xiang 2022).

Among the oldest and most common univariate measures of spatial dependency are Moran’s I and Geary’s C (Moran 1950, Geary 1954). Both rely on a similarity matrix reflecting spatial proximity between sample pairs and are applied to rank genes according to their scores (Hao *et al.* 2021). Under the null assumption of spatially independent standard normally distributed observations, both statistics follow a weighted sum of chi-squared distributions, which can be used to test and select individual features for spatial dependence (Cliff and Ord 1972). Moran’s I was later expanded by Wartenberg (1985) and Jombart *et al.* (2008) to measures of spatial dependence for multivariate data (multivariate spatial correlation, MSC, and spatial principal component analysis, sPCA), which in turn were used for dimension reduction.

While the machine learning literature is rich in methods that perform dimension reduction or feature extraction in a supervised setting, the development in the unsupervised setting lags behind (Solorio-Fernández *et al.* 2020). Principal component analysis (PCA) is still a common strategy. Among the more recent unsupervised feature selection methods, spectral filter methods have proven most successful in terms of performance and applicability to large data sets (Solorio-Fernández *et al.* 2020).

The seminal article by He *et al.* (2005) introduces the Laplacian score for the ranking of features. Starting from a matrix of pairwise sample similarities (e.g. cosine similarity or negative exponential of the squared Euclidean distance of the samples), it constructs the associated graph Laplacian and then calculates the squared length of a feature with respect to the norm induced by the graph Laplacian. It turns out that the Laplacian score is formally identical to Geary’s C when interpreting the similarity matrix as a spatial similarity matrix (Getis 1995). A notable extension of the Laplacian score is the SPEC score (Zhao and Liu 2007) which can be used to perform feature selection on both labelled and unlabelled data. The authors realized that the Laplacian score can be improved by spectral filtering. Spectral filtering represents a vector *x* of observations of a feature in the eigenbasis $\left\{v_{i};i=1,2,n\right\}$ of the graph Laplacian, $x=\sum_{i}\alpha_{i}\nu_{i}$. The coefficients of this decomposition are then re-weighted by some function $\gamma (\lambda_{i})$ of the corresponding eigenvalues $\lambda_{i}$, $x^{\prime }=\sum_{i}\gamma (\lambda_{i})\alpha_{i}\nu_{i}$. The SPEC score is defined as the squared length of the re-weighted vector. The weighting function γ puts more weight on eigenvectors with a strong spatial pattern, which are the low-frequency eigenvectors $\nu_{i}$ with a small eigenvalue. This includes classical low-pass filters which select only the most ‘relevant’ eigenvectors and discard all others (γ(λ)=1 if λ<c for some threshold value c, and 0 otherwise). Since then, there has been a surge of methods that suggest different similarity matrices (or an adaptive learning of them), different weighting schemes, and the replacement of the Laplacian score by more robust scores (Solorio-Fernández *et al.* 2020). In the unsupervised setting, to the best of our knowledge, the choice of the weighting function remains a matter of tuning, and the methods lack a proper statistical test for relevant features. The latter is largely because the similarity matrix needs to be constructed from the feature values, thereby confounding subsequent tests for feature relevance. This obstacle does not exist for spatial data, where the similarity matrix is calculated from the spatial layout of the experiment, but not the feature values.

In the present work, we express Moran’s I using a Hermitian operator on the space of expression patterns. Our algorithm, termed spatial component analysis (SPACO), constructs a spectral filter based on the eigenbasis of this operator. The threshold value of this filter is determined by a statistical test that does not involve manual choice and controls the family-wise error rate of the selected eigenvectors. We prove that the orthogonal projection of the data onto the space spanned by these eigenvectors maximizes Moran’s I, thereby pooling evidence of spatial dependence across genes with similar patterns. We show that this projection is a valid denoising operation for genes with a spatial pattern.

Under the assumption of independent, normally distributed feature values, the SPEC score corresponding to our filter follows a weighted sum of chi-squared distributions. Based on the tail probabilities of such a distribution we develop a statistical test for relevant features. We further show that this test is robust to violations of the normality assumption. Overall, SPACO achieves three goals in one natural framework: dimension reduction, feature selection, and denoising. We validate the performance of SPACO against state-of-the-art methods and show its favourable performance as a feature reduction method. For example, SPACO improves the clustering results of modularity-score optimization approaches such as Leiden clustering. The R implementation of SPACO and the complete data analysis code are provided in a Docker container available at https://github.com/IMSBCompBio/SpaCo, also accessible at https://doi.org/10.5281/zenodo.17965116.

## 2 Results

### 2.1 The SPACO algorithm

Suppose we want to analyse the spatial pattern of a feature x whose values have been measured at *n* spots with known locations, $x=(x_{i})\in R^{n}$. The spatial information is encoded as a non-negative, symmetric weight matrix $W=(w_{ij})\in R^{n\times n}$ with zero diagonal. The larger $w_{ij}$, the greater the spatial proximity of *i* and *j*. Moran’s I and Geary’s C are classical, widely used univariate measures of spatial dependence (Moran 1950, Geary 1954). For features *x* centred to mean 0 and scaled to variance 1, Moran’s I and Geary’s C take the form


$$I(x)=\frac{1}{\left|W\right|}\sum_{i,j=1}^{n}w_{ij}x_{i}x_{j}\;C(x)=\frac{1}{2|W|}\sum_{i,j=1}^{n}w_{ij}{\left(x_{i}-x_{j}\right)}^{2}$$

with $|W|=\sum_{i,j}w_{ij}>0$ (see Methods S1, available as supplementary data at *Bioinformatics* online). The weight matrix is often derived from a translation and rotation-invariant kernel applied to the physical distances of the spots in d dimensions, such as a Gaussian kernel (Smola and Schölkopf 1998). The weight matrix is typically chosen sparse, because this significantly accelerates calculations. In our applications, $w_{ij}$ is a simple delta function indicating whether nodes *i* and *j* are within a specified small distance (see Section 4 for details and alternative approaches).

The presented algorithm considers only Moran’s I, which is the more commonly used statistic. However, I(x)=1−C(x) for row-normalized weight matrices, and generally this equality holds approximately, meaning that optimizing Moran’s I and Geary’s C are essentially equivalent tasks. For a detailed discussion of this, including a PAC (probably approximately correct) bound for the difference between I(x) and 1−C(X), we refer to Methods S2, available as supplementary data at *Bioinformatics* online. Moran’s I typically assumes values between −1 and 1, with large values above 0 indicating spatially correlated expression, values of 0 indicating no spatial correlation and small values below 0 indicating spatial anticorrelation. Moran’s I measures, up to an additive constant, the squared length of a pattern with respect to a non-Euclidean, positive semidefinite scalar product. Specifically, let *E* be the identity matrix and $L=\frac{1}{n}E+\frac{1}{\left|W\right|}W$. Define the positive semidefinite scalar product ${\langle x,y\rangle }_{L}=x^{⊤}Ly$ and its induced norm $||x|{|}_{L}=\sqrt{{\langle x,x\rangle }_{L}}$. The key observation is that this norm is a meaningful measure of length in pattern space, because for centred and scaled patterns *x*,


$$\left||x|{|}_{L}^{2}={\langle x,x\rangle }_{L}=1+I(x\right)$$

Patterns with strong spatial dependency as measured by a large Moran’s I have greater length, while non-spatial patterns have shorter length. This motivated us to endow the pattern space $R^{n}$ with the above scalar product and its induced norm.

A data set consists of *n* samples that are *p* dimensional feature vectors that have been observed at known spatial positions. As features often are transcript abundances, we refer to them henceforth as genes. We represent the data as a spots × genes matrix $X=(x_{s,g})\in R^{n\times p}$, where $x_{s,g}$ denotes the abundance of gene g in spot *s*. For clarity and to avoid excessive notation, we consider one single matrix only. The extension to multiple spatial datasets is straightforward and is implemented in SPACO (Methods S3, available as supplementary data at *Bioinformatics* online).

An indispensable pre-processing step is PCA whitening (Kessy *et al.* 2018) of the data matrix *X*. After whitening, the columns of *X* have mean zero and *X* has identity covariance matrix (Section 4). The number of principal components kept is the minimum number to retain at least 95% of the variance of the data. To perform dimension reduction, SPACO performs a PCA of the data *X* with respect to the scalar product ${\langle .,.\rangle }_{L}$. Let $u_{1},u_{2},\ldots ,u_{p}\in R^{p}$ the eigenvectors of the covariance matrix $X^{⊤}LX$, sorted in decreasing order of their respective eigenvalues $\lambda_{1}\geq \lambda_{2}\geq \ldots \geq \lambda_{p}\geq 0$. We call the eigenvectors $u_{i}$ spatial components (SpaCs)*.* The corresponding patterns $v_{j}=Xu_{j}$ are called SpaC patterns. We orthogonally project the data onto the space $V_{k}=\langle v_{1},\ldots ,v_{k}\rangle$ for some *k*. Our procedure is motivated by the Courant–Fischer theorem, which implies that $V_{k}$ is optimal according to the following saddlepoint criterion (see Methods S4, available as supplementary data at *Bioinformatics* online, for a proof):


$$V_{k}=\underset{V\leq \langle X\rangle ,\dim(V)=k}{\text{argmax}}\;\underset{x\in V\backslash \{0\}}{\min}I(x)$$

**Figure 1:**
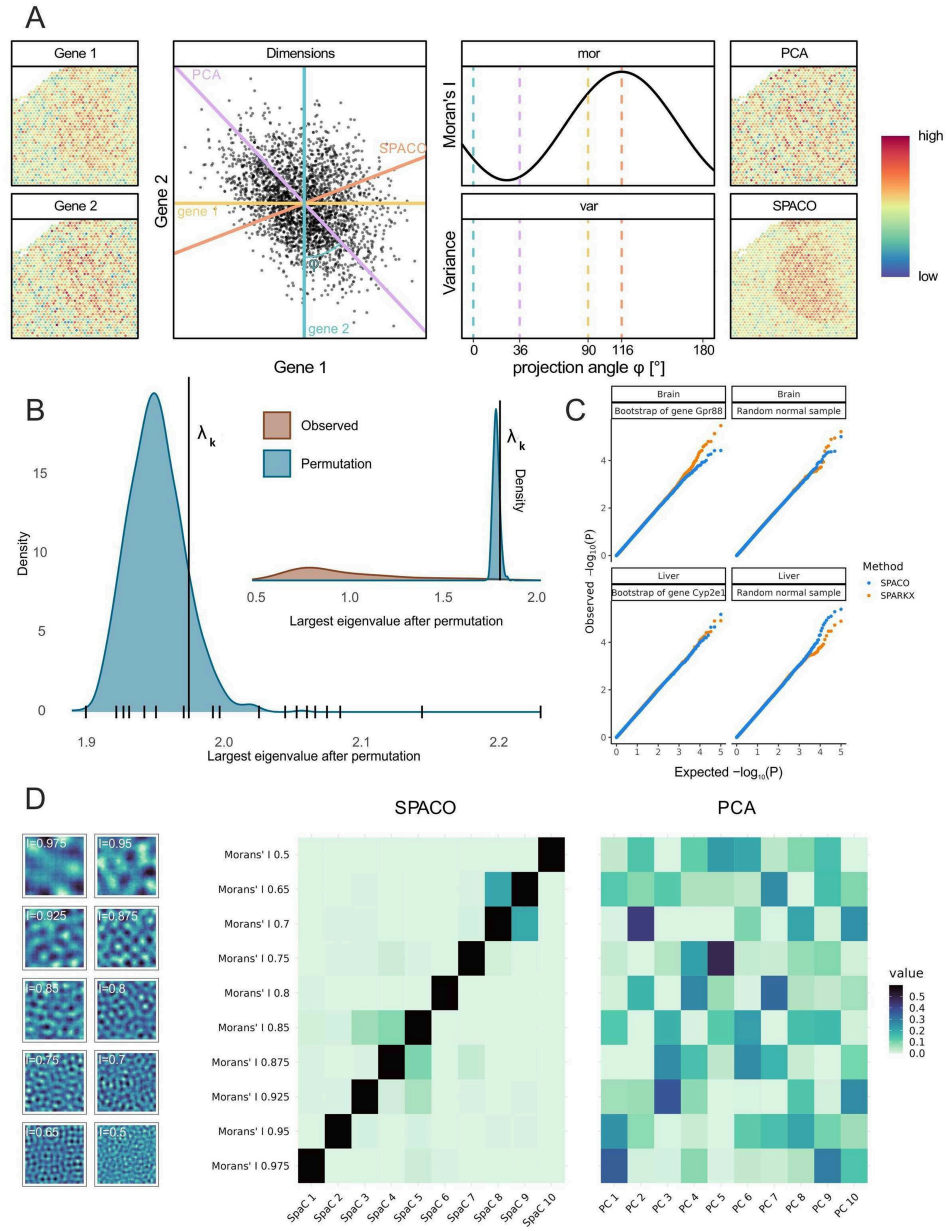
(A) Schematic of SPACO and PCA for two genes with weakly spatial patterns (left). Each spot carries two normalized expression values for Gene 1 and Gene 2, shown in the scatterplot. Coloured lines correspond to 1D subspaces onto which the data are projected orthogonally. For each projection, characterized by its angle with the *x*-axis, the variance and Moran’s I of the projected data is shown (top resp. bottom curve). Coloured dotted lines correspond to the projections of the same colour in the scatterplot. PCA chooses the projection to maximum variance as the first PC (top right), while SPACO chooses the projection with maximum Moran’s I as the first SpaC (bottom right). (B) Density of largest eigenvalue of ${\tilde{X}}^{⊤}L\tilde{X}$, where $\tilde{X}$ is a sampled random expression pattern of the brain dataset X after random spot permutation. The *x*-axis ticks are the eigenvalues of $X^{⊤}LX$. The vertical line at $\lambda_{k}$ shows the threshold for spatial components with relevant spatial information. The upper inset plot shows the density of the largest eigenvalue of permuted $Y^{⊤}LY$ (blue) and the eigenvalues of $X^{⊤}LX$ (orange). (C) Calibration plots of observed (*y*-axis) versus expected (*x*-axis) *P*-values from SPACO (blue) and SPARKX (orange) in the brain (top) and liver (bottom) data in two simulation settings using the null model (right) and random spot permutations of two genes with strong spatial patterns (Gpr88, Cyp2e1, left). (D) Application of SPACO respectively PCA to ten synthetic patterns with decreasing spatial structure (left, I = Moran’s I). Heatmaps show the squared loadings of each pattern with respect to each base vector (left: SPACO, right: PCA).

Put simply, projecting onto $V_{k}$ optimally preserves spatial patterns, in the sense that the ‘worst’ pattern contained in $V_{k}$ has maximal spatial dependency according to Moran’s I. An example for k=1 is given in Fig. 1A, where SPACO is contrasted to conventional PCA.

It remains to be decided which SpaC patterns show non-random spatial variation, i.e. we need to determine *k*. In PCA, one typically chooses *k* as the smallest integer such that the orthogonal projection onto $V_{k}$ captures at least a certain fraction of the total variance of the data. However, this approach does not guarantee the inclusion of all relevant components, nor can it guard against the inclusion of irrelevant components. However, with SPACO we can leverage the spatial arrangement of the data to make an informed decision. Consider the distribution of the largest eigenvalue of ${\tilde{X}}^{⊤}L\tilde{X}$, where $\tilde{X}$ is the data obtained from *X* by a random permutation of the spots. We consider all spatial components significant whose corresponding eigenvalue is greater than a certain quantile of that distribution (Fig. 1B, Section 4). We prove that our procedure controls the family-wise error rate (Methods S5, available as supplementary data at *Bioinformatics* online), which we set to 0.05 by default. As eigenvalue computations can be time-consuming for very large matrices, we derive an efficient approximation to the above test, which uses only a subset of all spots (Methods S6, available as supplementary data at *Bioinformatics* online). Once we bound the size of this subset, the algorithm has a runtime that is linear in the number of spots and the number of features.

Note that SPACO does not require any manual parameter choice. In the following, $P_{\text{SPACO}}$ will denote the orthogonal projection onto $V_{k}$, where *k* is the number of spatial components with a significant spatial pattern according to the above test.

### 2.2 SPACO gives rise to a well-calibrated test for spatially variable genes

We use the squared length of the projected pattern $P_{\text{SPACO}}(x)$ of a gene’s pattern *x* as a test statistic for spatial dependence. Under the null hypothesis that each spot of a non-spatial pattern *X* is sampled independently from a standard normal distribution, the null distribution of our test statistic becomes a weighted sum of chi-square distributions,


$$P_{\text{SPACO}}(X)∼\sum_{i=1}^{n}c_{i}\chi_{i}^{2}$$

where the coefficients $c_{i}$ are determined by the weight matrix and the data (Methods S7, available as supplementary data at *Bioinformatics* online). For a gene that has a non-random spatial pattern *x* (termed SVG) we expect that *x* is well approximated by $P_{\text{SPACO}}(x)$. Therefore, its projection $P_{\text{SPACO}}(x)$ should have greater length than expected by chance. Efficient algorithms to calculate the tail probabilities of our null statistic are available, we use the method of Davies (1980) implemented in the R package mgcv (Wood 2001). We verified that SPACO and a reference method, SPARKX, produce uniform *P*-values under this null model, paying particular attention to the lower end of the *P*-value distribution (Fig. 1C). Our test is robust against violations of the null model. We verified the calibration of the *P*-values when the data were not sampled from the null distribution, but from random spot permutations of SVGs (Fig. 1C). A pseudocode of the full SPACO dimension reduction and spatially variable feature testing algorithm is given in Methods S8, available as supplementary data at *Bioinformatics* online.

### 2.3 SPACO preserves and amplifies spatial information

Because there is no objective criterion to evaluate unsupervised dimensionality reduction methods, their usefulness is typically judged using surrogate measures. These include how well the method preserves relevant information from the original data and its impact on performance in downstream tasks within the spatial analysis pipeline. To assess information preservation, we simulated 10 expression patterns with decreasing spatial dependence (Fig. 1D, Methods S9 and S10, available as supplementary data at *Bioinformatics* online). A powerful dimension reduction method for spatial data should assign more importance to features with higher spatial dependence. In line with this expectation, the sequence of SpaCs aligns perfectly with the above sequence of features, while the sequence of principal components does not exhibit any such pattern (Fig. 1D).

### 2.4 SPACO enhances downstream tasks

**Figure 2:**
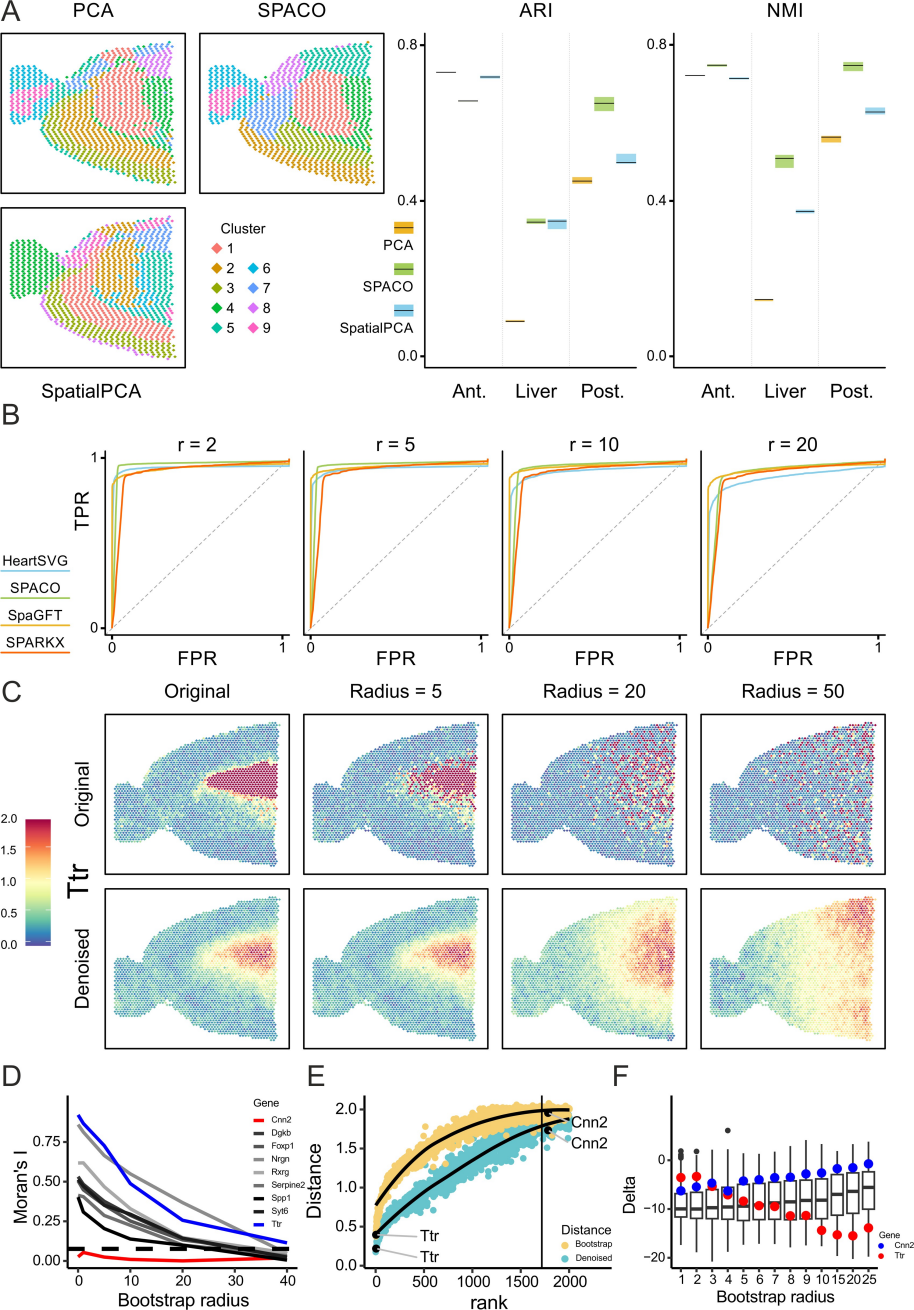
(A) Examples of Leiden clustering after dimension reduction with PCA (top left), SPACO (top right), and SpatialPCA (bottom). (B) ROC curves for HeartSVG (blue), SPACO (green), SpaGFT (yellow), and SPARKX (red) using the anterior brain data and 757 published spatial genes as cases and 757 randomly permuted copies as controls. Different ROC plots correspond to task difficulty, i.e. noise levels added to the cases. (C) Locally bootstrapped versions of Ttr, a gene with a strong spatial pattern (first row) and their respective projections (second row). Columns correspond to different radii of local resampling applied to the original measurements. (D) Decrease of Moran’s I with increasing levels of noise (radius 0 = original data, radius 40 = 75% of slide size) for a collection of genes, including Ttr (blue) and Cnn2 (red). Dotted line: median Moran’s I of randomly permuted patterns. (E) Distance (*y*-axis, RSS = average residual sum of squares per spot) of the original data to the perturbed patterns (radius = 5, yellow) and their denoised counterparts (turquoise), for the first 2000 genes ranked by significance (*x*-axis). The vertical line corresponds to an adjusted *P*-value of .05. (F) Distance reduction (*y*-axis, Delta = RSS denoised—RSS perturbed) achieved by denoising the set of significant SVG (boxes) for each radius (*x*-axis). The Delta values for Ttr (blue) and Cnn2 (red) are highlighted individually.

A typical downstream task for spatial data is spot clustering to detect functionally coherent regions. We, therefore, compared SPACO with PCA and SpatialPCA (Shang and Xiang 2022) with respect to their utility for clustering (Section 4). We applied Leiden clustering on two mouse brain datasets and one liver dataset. The SPACO clusters appear slightly more coherent and smoother (Fig. 2A). To systematically evaluate this, we split the spot grid into two adjacent spot sets shifted by one unit (Fig. S1A, available as supplementary data at *Bioinformatics* online). Each split dataset was processed and clustered completely independently. Considering each pair of adjacent spots as one locus, we obtain, for each method, two different clusterings of that locus. The consistency of the two clusterings was assessed by the adjusted rand index and by normalized mutual information (ARI and NMI, see Methods S12, available as supplementary data at *Bioinformatics* online, for the definition of all performance metrics used in this article, see also Fig. S1B, available as supplementary data at *Bioinformatics* online). SPACO performed best in all but one scenario (Fig. 2A). The performance was generally worst in the liver data, where the spatial structures are partly eliminated when the data were split. In conclusion, SPACO appears to be preferable over PCA and SpatialPCA for initial dimension reduction of multivariate spatial data.

### 2.5 Accurate SVG detection with SPACO

We developed a coverage-adjusted local resampling procedure to progressively diminish the spatial signal of a gene expression pattern while preserving its relative abundance distribution (Methods S10, Fig. S2, available as supplementary data at *Bioinformatics* online). Starting with the original pattern, we generate a new pattern by sampling values from the neighbourhood of each spot within a given radius *r*. The resampled pattern is then rescaled to match the total coverage per spot in the original data. This rescaling is essential, as unadjusted patterns show an artificial anticorrelation with the coverage pattern, which is itself correlated with biologically relevant patterns (Fig. S3, available as supplementary data at *Bioinformatics* online). This also highlights the inadequacy of unadjusted resampling techniques for SVG detection. Through ROC analysis, we compared SPACO’s specificity and sensitivity to three common SVG detection methods [SPARKX (Zhu *et al.* 2021), HeartSVG (Yuan *et al.* 2024), and SpaGFT (Chang *et al.* 2024]. The test data set consisted of 757 published spatial marker genes from the mouse brain sagittal-anterior dataset (10x Genomics, 2022) that were determined independently of the current dataset. These were paired with 757 non-spatial counterparts obtained via random resampling, serving as negative controls. The difficulty of detecting SVG was systematically increased by perturbing the original patterns of the spatial marker genes through coverage-adjusted local resampling with increasing radius *r* (Fig. 2B). SPACO consistently outperformed HeartSVG. Overall, SPACO had a higher sensitivity than the other methods across different levels of noise and had a smaller decrease in performance than SPARKX and HeartSVG. Notably, SpaGFT had the lowest change in performance over different noise levels. This might be explained by the fact that the Fourier modes, which can be thought of as SpaGFT’s equivalent to our spatial components, are computed directly from the coordinates, which do not change with the introduction of noise. For lower levels of noise and undistorted expressions, SPACO yielded higher AUC values than SpaGFT. It should further be noted that, if SPARKX is run on the unnormalized data as suggested in Zhu *et al.* (2021), it identifies about 15 000 significant SVG compared to the 1500–2500 of the other methods. The sensitivity of SPARKX to non-spatial genes is due to the use of unnormalized data. Without normalization, non-spatial genes tend to be anticorrelated with the spatial pattern of spot-wise coverage and are therefore tested significant (Methods S10, Fig. S3, available as supplementary data at *Bioinformatics* online).

### 2.6 SPACO is a denoising filter for spatially variable genes

Viewing SPACO as a filter method, we expect that the projection $P_{\text{SPACO}}$ acts as a denoising operation for expression patterns of SVG, while being a random projection for non-SVG. To assess this, we replaced each gene pattern by a noisy version of it using coverage-adjusted local resampling with a given radius *r* (see Fig. 2C). SPACO was performed on the resampled data, SVG was called, and the SPACO projection of each resampled pattern was compared to its original pattern that never entered the analysis pipeline (Fig. 2C). It turns out that the projections of the resampled SVG patterns are more similar to their unperturbed original patterns than the resampled SVG patterns themselves (Fig. 2D–F). This effect decreases as the spatial dependence of the patterns decreases, until the SPACO projection is no better than a random projection for non-SVG. In contrast, no similar denoising is observed when the SPACO projection is replaced by a projection onto the same number of principal components. It is worth noting that SPACO projections are spatial by construction for any input pattern (see the denoising of the Cnn2 gene in Fig. S2C, available as supplementary data at *Bioinformatics* online) and should therefore only be applied to patterns that are identified as significantly spatial. As a result, replacing SVG patterns with their SPACO projections improves their quality, with implications for interpretability and accuracy in subsequent tasks.

### 2.7 Application to biological data

Using SPACO on liver data, we recover genes with known periportal–pericentral hepatic lobule gradients (Cunningham and Porat-Shliom 2021), identifying 70% of previously detected genes. Undetected SVG (12 in total) were not found merely due to initial low coverage filtering by the SCTransform function of Seurat gribov2010seurat. GO-term analysis links SVGs to fatty acid metabolism and catabolic processes, key functions in periportal and pericentral zones. A similar 56% SVG recovery was achieved for anterior brain data. GO-term analysis of the genes identified as significant, but not listed in the literature, were associated with terms specific to the tissues analysed (Figure S4, available as supplementary data at *Bioinformatics* online).

## 3 Discussion

We have established SPACO as a robust and efficient tool for the analysis of high-dimensional spatial data. It addresses current limitations of existing methods by a dimension reduction which avoids arbitrariness through its automatic and statistically justified selection of dimensions. This makes SPACO particularly advantageous for downstream tasks such as spatial pattern testing and denoising of features with a spatial pattern. While many spatial pattern detection methods are highly sensitive, they often fall short in specificity. The robust and calibrated spatial pattern test provided by SPACO is a step forward in this regard.

SPACO is reasonably efficient; the analysis of a 10x Visium slide takes about 1 min on a standard laptop. SPACO’s ability to perform multi-slide analysis without requiring batch correction is another notable advantage. Note that a multi-slide analysis however preferentially detects features that exhibit spatial patterns across all slides, potentially overlooking features that are spatially variable only in specific conditions.

The only design choice we made in SPACO’s methodology is the construction of the neighbourhood weight matrix. We do this using a kernel function that assigns weights only to a few nearest neighbours. Based on the relationship I(x)≈1−C(x), and knowing that C(x) measures local variance relative to global variance, we hypothesise that the local variance estimate remains stable even with kernels of small width. This implies that SPACO achieves optimal sensitivity with locally focused kernels while maintaining sufficient robustness.

Looking ahead, the inclusion of additional features, such as incorporating directional derivatives [e.g. a Gabor filter (Singhal *et al.* 2024)] or curvature [e.g. a Laplacian filter (Bergenstråhle *et al.* 2020)], could further improve the robustness and accuracy of spatial pattern detection. Finally, applying SPACO as a dimension reduction or feature extraction method in unsupervised settings could provide probabilistic guarantees, expanding on the foundational work by He *et al.* (2005).

## 4 Methods

### 4.1 Data processing

Starting from a spots × genes matrix count matrix $X=(x_{s,g})\in R^{n\times p}$, we normalize the single cell RNA-seq data using SCTransform (Hafemeister and Satija 2019). If other spatial data modalities are being considered, this step should be replaced by other appropriate pre-processing methods. After *z*-transformation of each gene (centering and scaling the columns of *X* to mean 0 and variance 1), we perform PCA whitening on the SCTransformed data *X*. This is done by computing the empirical covariance matrix $C=\frac{1}{n}XX^{⊤}$ and its eigenvalue decomposition $C=EDE^{⊤}$, where the columns of *E* are the eigenvectors of *C* with eigenvalues given in the diagonal matrix $D=\text{diag}(d_{1},\ldots ,d_{p}),d_{1}\geq \ldots \geq d_{p}\geq 0$. To obtain data with identity covariance matrix, we apply the whitening transformation $X\to D^{-1/2}E^{⊤}X$. For reasons of efficiency, we only keep the minimum number of principal components necessary to retain at least 95% of the variance of the data. For convenience, the resulting matrix is again denoted as *X*.

### 4.2 Construction of the weight matrix

Matrix operations, such as multiplication, are significantly faster when performed on sparse matrices (matrices with relatively few non-zero entries). To leverage this efficiency, we construct a sparse neighbourhood weight matrix $W=(w_{ij})\in R^{n\times n}$ with at most *nN* non-zero entries, where *N* is a fixed number of nearest neighbours of a spot. For a rectangular grid used in many spatial transcriptomics applications, we include the nearest N=8 neighbours for each spot (except for the spots at the borders) and assign a weight of 1 to each edge between neighbours. For non-regular spot positions, we advise to identify the N=8 nearest neighbours $x_{j}$ of each spot $x_{i}$ and set $w_{ij}=1$ for these pairs of neighbours and $w_{ij}=0$ otherwise. Instead of choosing identical weights for these neighbours, one might consider a more nuanced weighting using some kernel function, say a Gaussian kernel $w_{ij}=K_{i}\;\text{exp}(\frac{-||x_{i}-x_{j}|{|}^{2}}{2\sigma_{i}^{2}})$. Here, $K_{i}$ is a normalizing constant such $\sum_{j}w_{ij}=1$. The width of this kernel may be tuned by choosing $\sigma_{i}$ such that the perplexity of the neighbourhood weights, $\text{Perp}(\sigma_{i})=2^{-\sum_{j}w_{ij}{\;\text{log}\;}_{2}(w_{ij})}=P$, equals some fixed constant P<N. For large *P*, this is equivalent to equal weighting, for small *P*, this will essentially pick only the nearest neighbour of *i*. The resulting, possibly asymmetric adjacency matrix $\tilde{W}$ is then symmetrized, leading to $W=\frac{1}{2}(\tilde{W}+{\tilde{W}}^{⊤})$.

### 4.3 Selection of relevant spatial components

To determine the dimension *k* of the space $V_{k}$ onto which SPACO projects the data, we need to determine which spatial components have a spatial pattern. To that end, let μ be the largest eigenvalue of the random matrix ${\tilde{X}}^{⊤}L\tilde{X}$, where $\tilde{X}$ is obtained by permuting the rows (spots) of *X* by a permutation π, and π has been sampled uniformly from all spot permutations. We draw an i.i.d. empirical sample $\mu_{1},\ldots ,\mu_{R}$ from μ. Given a fixed family-wise error rate γ (we choose γ=0.05), we construct a threshold *l* such that P(M>l)≤γ (Fig. 1B, Methods S5, available as supplementary data at *Bioinformatics* online). All spatial components $u_{j}$ whose eigenvalue $\lambda_{j}$ is greater than *l* are considered significant. We prove that this test procedure bounds the family-wise error rate by γ (Methods S5, available as supplementary data at *Bioinformatics* online).

### 4.4 Detection of spatially variable genes

The projection $P_{\text{SPACO}}$ decomposes a pattern *x* into the projection into SPACO space, $P_{\text{SPACO}}(x)$, and its *L*-orthogonal complement, $x-P_{\text{SPACO}}(x)$. For a gene that has a non-random spatial pattern (SVG), we expect that $x\approx P_{\text{SPACO}}(x)$, and therefore $P_{\text{SPACO}}(x)$ should be larger than to be expected by chance. To make this notion quantitative, our null model assumes that an expression pattern $Y\in R^{n}$ is spatially independent noise, Y∼N(0,I). Let $s_{i}=\frac{1}{\sqrt{\mu_{i}}}Xu_{i}\in {\mathbb{R}}^{n},i=1,\ldots ,n$, the *L*-orthonormalized expression patterns conjugate to the SpaCs $u_{i}\in R^{p}$ (note that ${\langle s_{i},s_{j}\rangle }_{L}=\frac{1}{\sqrt{\mu_{i}\mu_{j}}}u_{i}^{⊤}X^{⊤}LXu_{j}=\frac{1}{\sqrt{\mu_{i}\mu_{j}}}\delta_{ij}\mu_{j}=\delta_{ij}$ by construction). Then


(1) $$Y=\sum_{i=1}^{n}{\langle s_{i},Y\rangle }_{L}s_{i}=\sum_{i=1}^{n}\left(s_{i}^{⊤}LY\right)s_{i}.$$

Let $S_{k}=(s_{1},\ldots ,s_{k})\in R^{n\times k}$. The squared length of the *L*-orthogonal projection $P_{\text{SPACO}}(Y)$ of *Y* onto the first *k* SpaC patterns is


(2) $$\begin{array}{c} ||P_{\text{SPACO}}(Y)|{|}_{L}^{2}=||\sum_{i=1}^{k}\left(s_{i}^{⊤}LY\right)s_{i}|{|}_{L}^{2}=\sum_{i=1}^{k}{\left(s_{i}^{⊤}LY\right)}^{2}\\ =Y^{⊤}L^{⊤}S_{k}S_{k}^{⊤}LY=Y^{⊤}\Sigma Y \end{array}$$

With $\Sigma =L^{⊤}S_{k}S_{k}^{⊤}L$. By the spectral theorem, we decompose Σ into


(3) $$\Sigma =QCQ^{⊤}\in R^{n\times n}$$

with unitary matrix *Q* and diagonal matrix $C=\text{diag}(c_{1},\ldots ,c_{n})$. By rotational invariance of the standard multivariate normal distribution, $Z=Q^{⊤}Y∼N(0,I)$. It follows that $||P_{\text{SPACO}}(Y)|{|}_{L}^{2}$ is a weighted sum of independent chi-squared ($\chi^{2}(1)$) random variables,


(4) $$\begin{array}{cc} T:=||P_{\;\text{SPACO}}(Y)|{|}_{L}^{2}=Y^{⊤}\Sigma Y={\left(Q^{⊤}Y\right)}^{⊤}\cdot C\cdot (Q^{⊤}Y) & \\ =Z^{⊤}CZ=\sum_{i=1}^{n}c_{i}z_{i}^{2}∼\sum_{i=1}^{n}c_{i}\chi_{i}^{2} & \end{array}$$

with $\chi_{i}^{2}\underset{\text{iid}}{∼}\chi^{2}(1),i=1,\ldots ,n$. Computations can be accelerated slightly as the non-zero coefficients $c_{i}$ (of which there are at most *k*) can also be obtained as the eigenvalues of $\tilde{\Sigma }=(S_{k}^{⊤}L)(L^{⊤}S_{k})=S_{k}^{⊤}\text{diag}(\mu_{1}^{2},\ldots ,\mu_{k}^{2})S_{k}\in R^{k\times k}$. Note that the column vectors in $S_{k}=(s_{1},\ldots ,s_{k})$ are not orthogonal in Euclidean space, and therefore $\tilde{\Sigma }$ is in general not a diagonal matrix.

Given a gene *g* with corresponding expression pattern $x_{g}$ (centered to mean 0 and variance 1), our test statistic is


(5) $$t_{g}\text{:}=||P_{\text{SPACO}}(x_{g})|{|}_{L}^{2}=x_{g}^{⊤}\Sigma x_{g}$$

and we test the one-sided hypothesis $p_{g}\text{:}=P(T\geq t_{g})$ using the null distribution in Equation (4), see Methods S7, available as supplementary data at *Bioinformatics* online.

### 4.5 *P*-value calibration

We checked the calibration of SPACO’s SVG test (i.e. uniform distribution of *P*-values under the null hypothesis) and another state-of-the-art method, SPARKX (Zhu *et al.* 2021). Note that the coefficients $c_{i}$ in SPACO’s test statistic (Equation 5) depend on the data. We, therefore, validated the SVG test separately in the anterior brain and the liver datasets. We calculated the respective coefficients $c_{i}$ and sampled $10^{5}$ expression patterns from our null model (independent, standard normally distributed values per spot). After calculating the corresponding *P*-values, we compared the empirical *P*-value distribution with the expected (uniform) *P*-value distribution, both for SPACO and for SPARKX.

To further check robustness against violations of the null model, we applied the same procedure to $10^{5}$ random patterns obtained from the spatial genes Gpr88 (brain) and Cyp2e1 (liver) by coverage-adjusted, unrestricted resampling. Main Fig. 1C confirms that both methods produce well-calibrated *P*-values in all four scenarios.

### 4.6 Split dataset analysis and clustering comparison

For assessing the consistency of clustering, each of the three datasets was divided into two by splitting the spot grid into pairs of mutually adjacent spots that are shifted horizontally by one unit (Fig. S1A, available as supplementary data at *Bioinformatics* online). The left (right) spot of each pair was assigned to grid 1 (grid 2). Some points at the left/right margins that did not have a partner were discarded. This resulted in a pair of artificial datasets for each sample. The liver datasets had 777 spots per group, the anterior mouse brain dataset 1319 spots per group, and the posterior mouse brain dataset 1652 spots per group. Each of these datasets was then processed independently using either the Seurat (V. 5.3.1) standard pipeline for 10X Visium spatial data analysis (Hao *et al.* 2024), the SpatialPCA pipeline described in Shang and Xiang (2022), or the standard SPACO analysis pipeline. Upon completion of processing, the calculated significant spatial components were integrated into the pre-existing Seurat objects via Seurat’s CreateDimReducObject function. For contrasting the spatial component clustering result with the PCA-based clustering, PCA was performed on the two datasets per sample utilizing the RunPCA function of Seurat. The number of principal components employed for clustering was determined through an elbow plot of the principal components. The neighbourhood graph for clustering was computed with the FindNeighbors function, using either the significant principal components or spatial components. Leiden clustering was executed through the Seurat’s FindClusters function. To ensure a fair comparison, the resolution parameters were modified to yield the same number of clusters for both original datasets and all methods (the agreement of clusterings becomes increasingly fragile with increasing number of predicted clusters). The clustering agreement between two split datasets was measured by the adjusted Rand index (ARI) and the normalized mutual information (NMI). ARI was calculated using the mclust (V. 6.0.0) package’s adjustedRandIndex function (Scrucca *et al.* 2016). NMI was calculated with the aricode (V. 1.0.2.) package, using the ‘max’ variant of the NMI function (CRAN—Package aricode, n.d.). As Leiden clustering starts with a random initiation, the process was repeated with 1000 different random seeds. Results illustrated in Fig. 1C are derived only from those paired datasets that resulted in the identical number of clusters for both splits.

### 4.7 Assessment of the denoising property of the SPACO projection

For the evaluation shown in Fig. 2D–F, we performed coverage-adjusted local resampling of the datasets for the depicted radii. All patterns were projected *L*-orthogonally onto the relevant SpaCs. The delta values in Fig. 2E were calculated as the squared Euclidean distance between the original pattern and the denoised pattern, divided by the number of spots (regarding the denoised pattern as an approximation of the original, this is the average squared residual per spot). For Fig. 2F, we calculated the Euclidean distance of all SVG per radius from the resampled gene to the original and from the denoised gene to the original.

### 4.8 Spatially variable gene detection benchmarking

To assess the capacity of SPARKX and SPACO to identify SVGs at various noise levels, we used a gold standard of 757 spatial marker genes that have been previously documented for the mouse brain anterior dataset (Bae *et al.* 2022), available in the Supplementary Data, available as supplementary data at *Bioinformatics* online. For each gene in this collection, we added a non-spatial ‘twin’ gene as a negative control, which was obtained from the gene’s expression pattern by coverage-adjusted, unrestricted resampling. All other genes merely served as background data. Subsequently we increased the difficulty of the SVG detection task step by step and generated ten individual benchmarking datasets that were identical to the previous one, except for the 757 marker genes. The spatial pattern of those was blurred by coverage-adjusted local resampling, for increasing radii (*r *= 2, 5, 10, 20, see Fig. 1E). Using the 757 marker genes as cases and their randomly permuted twins as controls, we calculated a receiver operating characteristic (ROC) curve for each dataset based on the SVG test of SPACO and SPARKX. For each scenario and each test method, we generated 10 independent datasets. We report a median ROC curve for each scenario and method using the median of the 10 sensitivity values for a given specificity. For SVG testing with SPARKX, we followed the protocol in Zhu *et al.* (2021). In particular, all genes with zero counts were removed, the coordinates were normalized to a mean of 0 and a variance of 1, and the genes were similarly adjusted as described in the methods section of SPARKX. The ‘mixture’ option was selected for kernel choice. For HeartSVG, we followed the software documentation referenced in Yuan *et al.* (2024). Minimal adaptations were necessary in the data construction scripts to enable use of HeartSVG with the current Seurat version. The SpaGFT analysis was performed as suggested in Chang *et al.* (2024). Python version 3.8.20 was used. Count data was saved as csv to enable benchmarking across R and python.

## 5 Experimental data analysis

### 5.1 10X Visium brain datasets

The Visium spatial transcriptomics dataset for the mouse brain was downloaded from the 10X Genomics Data Repository. The mouse brain serial anterior section slide contains 2695 spots and 32 285 genes, and the posterior slide contains 3353 spots and 31 053 genes. Sequencing metrics can be found in the 10X Genomics Data repository https://www.10xgenomics.com/resources/datasets (10x Genomics, 2022).

### 5.2 10X genomics Visium liver data

The spatial transcriptomics dataset for young mouse liver was taken from Nikopoulou *et al*. (2023). It contains 1596 spots and 32 285 genes. Sequencing metrics and sample preparation details can be found in the original publication. The GO-term analysis shown in Fig. 2B and C and Fig. S4, available as supplementary data at *Bioinformatics* online, were done using Clusterprofiler (V. 4.2.2.) (Yu *et al.* 2012) using the standard parameters and ‘biological process’ as term input. Gene lists for the comparison of SVGs detected using SPACO and described in the literature were obtained from Bae *et al.* (2022) for the brain dataset and from Hildebrandt *et al.* (2021) for the liver dataset.

### 5.3 Code and reproducibility

All panels shown in Figure 2 can be reproduced using the R code and the data provided in the Docker container available at https://github.com/IMSBCompBio/SpaCo.

## Supplementary Material

btag052_Supplementary_Data
