# Supplementary material for: A spectral dimension reduction technique that improves pattern detection in multivariate spatial data
Source: Bioinformatics. 2026 Jan 31;42(2):btag052. doi: 10.1093/bioinformatics/btag052 (PMC12925250; doi:10.1093/bioinformatics/btag052)
Supplement: btag052_Supplementary_Data [file btag052_supplementary_data.zip › SPACO_Supplemental_Methods.pdf]

A spectral Dimension Reduction technique That improves Pattern Detection in multivariate spatial Data

David Köhler, Niklas Kleinenkuhnen, Kiarash Rastegar, Till Baar, Chrysa Nikopoulou, Vangelis Kondylis, Vlada Milchevskaya, Matthias Schmid, Peter Tessarz, and Achim Tresch

S1 Moran's I and Geary's C, Introduction and Notation

In the following, we assume to have observed a set of p genes for a set of n spots. The data is represented as a spots $\times$ genes matrix $X = (x_s^g) \in \mathbb{R}^{n \times p}$, where x_s^g denotes the expression of gene g in spot s. For a gene g, we call the column vector $x^g = (x_s^g)_{s=1,\ldots,n}$ its expression pattern. Generally, we call any vector $x \in \mathbb{R}^n$ an expression pattern. Two expression patterns that are identical up to linear transformation are considered biologically indistinguishable. We therefore standardize the gene expression patterns x^g, i.e., we center them to mean zero and scale to variance one. For convenience, we recall the general formula for Moran's I and Geary's C and their simplified form used here.

Let $W = (w_{ij}) \in \mathbb{R}^{n \times n}$ is a non-negative weights matrix with zero entries in the diagonal, and $|W| = \sum_{i,j=1}^{n} w_{ij} > 0$. A positive value of w_{ij} is meant to indicate proximity/neighborhood of locus i and j, while $w_{ij} = 0$ indicates that i and j are not in proximity to each other. Let $\bar{x} = \frac{1}{n} \sum_i x_i$ the empirical mean of the univariate data $x \in \mathbb{R}^n$. Moran's I, $I(x)$, and Geary's C, $C(x)$ are defined as

$$I(x) = \frac{\frac{1}{|W|} \sum_{i,j} w_{ij}(x_i - \bar{x})(x_j - \bar{x})}{\frac{1}{n} \sum_i (x_i - \bar{x})^2} \quad (1)$$

$$C(x) = \frac{\frac{1}{2|W|} \sum_{i,j} w_{ij}(x_i - x_j)^2}{\frac{1}{n-1} \sum_i (x_i - \bar{x})^2} \quad (2)$$

By convention, summation over i and j will henceforth be from 1 to n. We set $M(x) = -\infty$ and $C(x) = \infty$ whenever the denominator in (2) is zero, i.e. when x is a constant vector.

We henceforth assume without loss that $|W| = n$, because Moran's I, and Geary's are invariant to proportional scaling of W. If the vector x has mean 0 and variance 1 (which implies $\|x\|_2 = 1$), the two expressions above simplify to

$$I(x) = \frac{1}{n} \sum_{i,j=1}^{n} w_{ij} x_i x_j \quad (3)$$

$$C(x) = \frac{1}{2n} \sum_{i,j=1}^{n} w_{ij}(x_i - x_j)^2 \quad (4)$$

Further, letting $L = \frac{1}{n}E + \frac{1}{|W|}W$ (E the identity matrix),

$$1 + I(x) = x^T L x =: \|x\|_L^2 \quad (5)$$

S2 Construction of the spatial weight matrix and asymptotic equivalence of $C(x)$ and $1 - I(x)$

Let $A = (A_{ij})_{i,j=1}^n$ be the symmetric and non-negative proximity weights matrix with $A_{ii} = 0$. Define the row-normalized matrix B,

$$s_i = \sum_{j=1}^{n} A_{ij} \ , \quad D = \mathrm{diag}(s_1, \ldots, s_n), \qquad B = D^{-1}A.$$

Then B is row-stochastic: $\sum_j B_{ij} = 1$. We symmetrize B to obtain a symmetric weights matrix W,

$$W = \tfrac{1}{2}(B + B^\top)$$

Since $\sum_{i,j} B_{ij} = n$, we have $\sum_{i,j} w_{ij} = n$.

We assume there exist constants $0 < s_{\min} \leq s_{\max} < \infty$, independent of n , such that $s_i \in [s_{\min}, s_{\max}]$ for all i. This condition is fulfilled by many natural choices of A . For example, if A is a k-nearest neighbours kernel, or if A is an Gaussian kernel on a regular grid, this property follows immediately.

Define the column sums of B by

$$c_i = \sum_{j=1}^{n} B_{ji}$$

Note that the row sums d_i of W can be expressed in terms of c_i ,

$$d_i = \sum_{j=1}^{n} w_{ij} = \frac{1}{2}\sum_{j=1}^{n}(B_{ij} + B_{ji}) = \frac{1}{2}\left(\sum_{j=1}^{n} B_{ij} + \sum_{j=1}^{n} B_{ji}\right) = \tfrac{1}{2}(1 + c_i)$$

since $\sum_j B_{ij} = 1$.

Because Moran's I and Geary's C are invariant under translation and scaling, we assume without loss that the pattern $x = (x_1, \ldots, x_n)$ is centred and standardised so that

$$\frac{1}{n}\sum_{i=1}^{n} x_i = 0 \ , \quad \frac{1}{n}\sum_{i=1}^{n} x_i^2 = 1$$

Define $\Delta = C(x) - (1 - I(x))$. Then, using Equations (3) and (4),

$$\begin{aligned}
\Delta &= \frac{1}{n}\sum_{i,j=1}^{n} w_{ij}x_i x_j + \frac{1}{2n}\sum_{i,j=1}^{n} w_{ij}(x_i - x_j)^2 - 1 \\
&= \frac{1}{n}\sum_{i=1}^{n} d_i x_i^2 - 1 \\
&= \frac{1}{n}\sum_{i=1}^{n}(d_i - 1)x_i^2
\end{aligned}$$

In particular, $\Delta = 0$ if $d_i = 1$ for all i , so the comparison between $C(x)$ and $1 - I(x)$ reduces to controlling d_i .

Using symmetry of A ,

$$c_i = \sum_{j=1}^{n} B_{ji} = \sum_{j=1}^{n} \frac{A_{ji}}{s_j} = \sum_{j=1}^{n} \frac{A_{ij}}{s_j}$$

By $s_j \in [s_{\min}, s_{\max}]$ we obtain

$$\frac{s_i}{s_{\max}} = \frac{1}{s_{\max}} \sum_{j=1}^{n} A_{ij} \le c_i \le \frac{1}{s_{\min}} \sum_{j=1}^{n} A_{ij} = \frac{s_i}{s_{\min}}$$

Using again $s_i \in [s_{\min}, s_{\max}]$ gives the uniform bound

$$\frac{s_{\min}}{s_{\max}} \le c_i \le \frac{s_{\max}}{s_{\min}} \quad \text{for all } i.$$

Therefore

$$|d_i - 1| = \tfrac{1}{2}|c_i - 1| \le \tfrac{1}{2} \max\left\{1 - \frac{s_{\min}}{s_{\max}}, \frac{s_{\max}}{s_{\min}} - 1\right\} =: M$$

where $M < \infty$ depends only on the ratio $s_{\max}/s_{\min}$.
To prove the convergence $\Delta \to 0$ in probability and a PAC bound, let $x_1, \ldots, x_n$ be i.i.d.\ with $E[X^2] = 1$. We further need to assume that X has a finite fourth moment, $E[X^4] < \infty$.
Then $E(x_i^2) = 1$ and $\mathrm{Var}(x_i^2) = \mathrm{Var}(X^2) < \infty$.
From $\sum_i (d_i - 1) = \sum_{ij} w_{ij} - n = 0$ we have $E(\Delta) = 0$. By independence of the x_i ,

$$\mathrm{Var}(\Delta) = \mathrm{Var}\left(\frac{1}{n}\sum_{i=1}^{n}(d_i - 1)x_i^2\right) = \frac{1}{n^2}\sum_{i=1}^{n}(d_i - 1)^2 \,\mathrm{Var}(X^2) \le \frac{M^2 \,\mathrm{Var}(X^2)}{n}$$

Hence $\mathrm{Var}(\Delta) \to 0$ as $n \to \infty$, and Chebyshev's inequality implies that for any $\varepsilon > 0$,

$$\mathbb{P}(|\Delta| > \varepsilon) \le \frac{\mathrm{Var}(\Delta)}{\varepsilon^2} \le \frac{M^2 \,\mathrm{Var}(X^2)}{n\,\varepsilon^2} \xrightarrow[n\to\infty]{} 0$$

so $\Delta \to 0$ in probability. Equivalently, $C(x) - (1 - I(x)) \to 0$ in probability. Moreover, the same inequality gives a finite-sample PAC bound: for any $\delta \in (0,1)$,
with probability at least $1 - \delta$,

$$|\Delta| \le M\sqrt{\frac{\mathrm{Var}(X^2)}{n\,\delta}}, \quad \text{i.e.} \quad \big|\,C(x) - (1 - I(x))\,\big| \le M\sqrt{\frac{\mathrm{Var}(X^2)}{n\,\delta}}$$

□

S3 Application of SPACO to multiple datasets

SPACO can be applied simultaneously to a number of datasets, e.g. a set of slides, in a straightforward manner. Given t slides with $n_1, ..., n_t$ spots. When processing multiple slides at a time, we scale each pattern $x_k \in \mathbb{R}^{n_K}$ to mean 0 and variance 1. Let $n = n_1 + ... + n_t$, and let $x = (x_1^T, ..., x_t^T)^T \in \mathbb{R}^n$ be the combined pattern of all slides. Given neighborhood matrices $W_1, ..., W_t$, $W_k \in \mathbb{R}^{n_k \times n_k}$, the joint spot space $\mathbb{R}^n = \mathbb{R}^{n_1} \times ... \times \mathbb{R}^{n_t}$ can be endowed with a scalar product inherited from the individual spot spaces,

$$\langle x^T, y \rangle_L = \sum_{k=1}^{t} \alpha_k x_k^T L_k y_k = x^T L y \quad , \quad x = (x_1^T, ..., x_t^T)^T, y = (y_1^T, ..., y_t^T)^T \in \mathbb{R}^n \tag{6}$$

with positive weights α_k and $L_k = \frac{1}{n_k} E_{n_k} + \frac{1}{|W_k|} W_k$ (E_{n_k} being the n_k-dimensional unit matrix) and $L = \text{diag}(\alpha_1 L_1, ..., \alpha_t L_t)$. There are two natural choices for α_k: Either, we set $\alpha_k = |W_k|$ and put equal weight on all pairwise observations, no matter from which slide. Or, by default, we set $\alpha_k = 1$ and assign equal weight to all slides (thereby emphasizing the equal importance of each sample). Thus, to perform SPACO on multiple datasets $X = \begin{pmatrix} X_1 \\ \vdots \\ X_t \end{pmatrix} \in \mathbb{R}^{n \times p}$ simultaneously, we perform the eigenvalue decomposition of the matrix $X^T L X = \sum_{k=1}^{T} \alpha_k X_k^T L_k X_k$.

S4 The SPACO projection satisfies a saddlepoint (min-max) criterion

Let the data after whitening transform be represented as a spots $\times$ genes matrix $X = (x_s^g) \in \mathbb{R}^{n \times p}$, and let $L = \frac{1}{n}E + \frac{1}{|W|}W \in \mathbb{R}^{n \times n}$ the matrix defining our scalar product. Let $u_1, ..., u_p \in \mathbb{R}^p$ be the eigenvectors of the generalized covariance matrix $X^T LX = \left(\left\langle x^g, x^h \right\rangle_L\right)_{g,h=1,...,p}$ corresponding to the eigenvectors $\lambda_1 \geq \lambda_2 \geq ...\lambda_p \geq 0$. Fix some $k \in \{1, ..., p\}$. The Courant-Fisher theorem (also known as min-max principle), applied to the matrix $X^T LX \in \mathbb{R}^{p \times p}$, states that

$$\lambda_k = \max_{U \leq \mathbb{R}^p,\, \dim U = k} \min_{u \in U \setminus \{0\}} \frac{u^T X^T LXu}{u^T u} \tag{7}$$

and the value λ_k is achieved for $U = U_k := \text{span}\,\langle u_1, ..., u_k \rangle$.

Let $V \leq \left\langle x^1, ..., x^p \right\rangle \leq \mathbb{R}^n$, $\dim V = k$, for some $k = 1, ..., p$. Then there exists some $U \leq \mathbb{R}^p$, $\dim U = k$, such that $V = XU$. We have

$$\begin{aligned} \lambda_k \overset{(7)}{\geq} \min_{u \in U,\, \|u\|_2 = 1} \|Xu\|_L^2 &\overset{(5)}{=} 1 + \min_{u \in U,\, \|u\|_2 = 1} I(Xu) \\ &\overset{(*)}{=} 1 + \min_{v \in V,\, \|v\|_2 = 1} I(v) \\ &= 1 + \min_{v \in V \setminus \{0\}} I(v) \end{aligned} \tag{8}$$

Equations (*) above holds because $\{Xu \mid u \in U, \|u\|_2 = 1\} = \{v \in V, \|v\|_2 = 1\}$. This is true because X is white by assumption, and $V = XU$. Hence. The last equation in (8) holds because Moran's I is invariant to multiplicative scaling.

On the other hand, letting $V_k = XU_k$,we have

$$\lambda_k = \min_{u \in U_k,\, \|u\|_2 = 1} \|Xu\|_L^2 \overset{(8)}{=} 1 + \min_{v \in V_k,\, \|v\|_2 = 1} I(v) = 1 + \min_{v \in V_k \setminus \{0\}} I(v) \tag{9}$$

Combining (8) and (9) yields the saddlepoint criterion stated in the main text,

$$\lambda_k = \max_{V \leq \langle x^1, ..., x^p \rangle, \dim V = k} \min_{v \in V \setminus \{0\}} I(v) \tag{10}$$

$$V_k = \operatorname*{argmax}_{V \leq \langle x^1, ..., x^p \rangle,\, \dim V = k} \min_{v \in V \setminus \{0\}} I(v) \tag{11}$$

$\square$

S5 Selection of relevant spatial components with control of the family-wise error rate

We will make use of a well-known method for the construction of non-parametric confidence intervals for the quantiles of a real-valued random variable, see e.g. [1]. For the reader's convenience, we adapt the construction to our notation.

Lemma. Let M_1 be a real-valued random variable, and let $\mu_1, ..., \mu_R$ be i.i.d. samples from M_1. Let $\mu_{(1)} \leq \mu_{(2)} \leq ... \leq \mu_{(R)}$ the samples ordered by size, and let $\mu_{(R+1)} = \infty$.

a) For given $\alpha, \beta \in (0, 1)$, let $l = l(\alpha, \beta)$ the smallest integer such that for a binomial random variable $B \sim \text{Bin}(R, 1-\alpha)$, we have $P(B > l-1) \leq \beta$ (or, equivalently, $P(B \leq l-1) > 1-\beta$. Then,

$$P(M_1 \geq \mu_{(l)}) \leq \alpha + \beta \tag{12}$$

b) For a given $\gamma \in (0, 1)$, let

$$l = \min \{l(\alpha, \beta);\ \alpha, \beta > 0,\ \alpha + \beta = \gamma\} \tag{13}$$

Note that l merely depends on γ, but not on M_1. Then, letting $r = \mu_{(l)}$, we have

$$P(M_1 \geq r) \leq \gamma \tag{14}$$

Proof. Let q be a $(1-\alpha)$-quantile of M_1. The probability that a sample μ_j is less than or equal to q is $1-\alpha$. Consequently, the number B of samples $\mu_1, ..., \mu_R$ that are less than or equal to q is bounded from above by a binomial distribution, $\text{Bin}(R, 1-\alpha)$. Further, $q \geq \mu_{(l)}$ holds exactly if more than $l-1$ samples are smaller or equal to q, for $l = 1, ..., R+1$. This implies $P(q \geq \mu_{(l)}) = P(B > l-1)$. It follows that

$$\begin{aligned} P(M_1 \geq \mu_{(l)}) &= 1 - P(M_1 < \mu_{(l)}) \\ &\leq 1 - P(M_1 < q \wedge q < \mu_{(l)}) \\ &= P(M_1 \geq q \vee q \geq \mu_{(l)}) \\ &\leq P(M_1 \geq q) + P(q \geq \mu_{(l)}) \\ &= \alpha + P(B > l-1) \\ &\leq \alpha + \beta \end{aligned} \tag{15}$$

b) follows from a), because $l_\gamma = l(\alpha, \beta)$ for some $\alpha, \beta > 0,\ \alpha + \beta = \gamma$. □

To determine the dimension k of the space V_k onto which SPACO projects the data, we need to determine which SpaCs v_i, $i = 1, ..., p$, are spatial. To that end, we test whether eigenvalue μ_i corresponding to v_i is significantly larger than to be expected by chance. Let $\lambda = (\lambda_1, ..., \lambda_p) = \text{spec}(X^T L X)$, $\lambda_1 \geq \lambda_2 \geq ... \geq \lambda_p$, the ordered sequence of eigenvalues corresponding to the SpaCs in the data. Let the random variable $M = (M_1, ..., M_p)$, $M_1 \geq M_2 \geq ... \geq M_p$ contain the sorted eigenvalues of the SpaCs when the data is drawn from the permutation null model, i.e.,

$$M \quad \sim \quad \text{spec}(\tilde{X}^\top L \tilde{X}) \tag{16}$$

Here, $\tilde{X}$ is the random matrix obtained by permuting the rows of X by a random permutation π, and π is sampled uniformly from all spot permutations. The sequence of hypotheses we want to test is $H_j : M_j \geq \lambda_j$, $j = 1, ..., p$. We derive a test procedure that controls the family-wise error rate at a specified level γ (by default, we let $\gamma = 0.05$). We draw a i.i.d. sample $\mu = (\mu_1, ..., \mu_R)$ from M_1 (the sitze of R will be determined later). Let l as constructed in the above lemma and $r = \mu_{(l)}$ the l-th smallest value in μ. By the above lemma, $P(M_1 \geq r) \leq \gamma$. Now choose $k = \max\{i;\ \lambda_i \geq r\}$ and reject $H_1, ..., H_k$. Note that $M_j \geq \lambda_j$ for some $j \in \{1, ..., k\}$ implies $M_1 \geq M_j \geq \lambda_j \geq \lambda_k$. It follows that the family-wise error rate for the first k hypotheses, $P(\cup_{i=1}^k H_j)$, is bounded by γ:

$$\begin{aligned} P(\cup_{i=1}^k H_j) &= P(M_j \geq \lambda_j \text{ for at least one } j \in \{1, ..., k\}) \\ &\leq P(M_1 \geq \lambda_k) \\ &\leq P(M_1 \geq r) \\ &\leq \gamma \end{aligned} \tag{17}$$

The last inequality holds by the above Lemma. □

In our implementation we start with $R = 50$ empirical samples of M_1 to construct k. We then increase the sample size in steps of 10 and repeat the construction of k until the sequence of k's obtained this way becomes constant for 5 iterations. To avoid excessive computations, we also stop when the maximum number of iterations (1000 by default) is reached.

S6 Efficient eigenvalue sampling

For large spot number n and feature number p, the eigenvalue computation of $\tilde{X}^\top L\tilde{X}$ can be expensive. To reduce the computational burden, we use an efficient approximation based on subsampling spots. Let $m \ll n$ be the target number of spots in the subsample. We iteratively sample a spot i uniformly at random and also include its neighboring spots j, $W_{ij} \neq 0$. This is repeated until we have drawn a set M containing at least m spots. Reducing the data to $X_m = (x_{i.})_{i\in M}$, the reduced neighborhood matrix becomes $W_m = (W_{ij})_{i,j\in M}$, and the operator on this reduced graph becomes $L_m = \frac{1}{m}E_m + W_m$. The sampling from the distribution of largest eigenvalues is then performed using X_m and L_m instead of X and L.

While this is a simple and fast solution, a more principled approximation with accuracy guarantees follows the logic of geometric multigrid. We construct a hierarchy of coarser graphs as follows. Partition the spatial domain into a regular grid (in 2D, a square grid) such that each cell contains approximately k points, say $k = 4$. Replace the points in each cell by their average feature vector, and define a coarse weighted graph on the cells by summing the original edge weights between points in different cells. This aggregation step is efficient because the weight matrix is sparse. Repeat this coarsening procedure to obtain several levels, each with fewer nodes. On the coarsest level, solve the eigenvalue problem using the Lanczos algorithm (or another Krylov subspace method). Then use this solution to initialize and precondition the eigenvalue problem on the next finer grid, and proceed level by level up to the original resolution. This yields a fast multilevel solver for the largest eigenvalue and eigenvector. The same multilevel procedure must be applied to each permuted matrix $\tilde{X}$, but the computation over permutations is easily parallelized.

S7 A test for detecting spatially variable genes using the SPACO projection

Let V a k-dimensional subspace of $\mathbb{R}^B$, where B is a subset of the set S of all spots. Let $\langle x, y\rangle = x^T L y$ the scalar product on $\mathbb{R}^B$ (note that L needs to be defined suitably with respect to the spot space B and differs from a scalar product defined on the entire spot space S). Let P the orthogonal projection onto V, and let V be spanned by the orthonormal column vectors $v_1, ..., v_k \in \mathbb{R}^B$. For a pattern $x \in \mathbb{R}^B$,

$$Px = \sum_{i=1}^{k} \langle x, v_i\rangle\, v_i = \sum_{i=1}^{k} x^T L v_i \cdot v_i \tag{18}$$

As our test statistic we choose the squared length of Px as measured by our scalar product, $\|Px\|_L^2$. Our null hypothesis is that the entries of a non-spatial random pattern X are i.i.d. standard normal samples, $X \sim \mathcal{N}(0, \mathrm{id})$. The null distribution of our test statistic can be calculated as follows: Let $L \sum_{i=1}^{k} \left(v_i {v_i}^T\right) L = R^T D R$ with unitary matrix R and diagonal, non-negative matrix $D = \mathrm{diag}(d_1, ..., d_S)$. Then

$$\begin{aligned} T &= \|PX\|_L^2 \\ &= \sum_{i=1}^{k} (X^T L v_i)^2 \\ &= X^T L \sum_{i=1}^{k} (v_i v_i^T) L X \\ &= X^T R^T D R X \\ &\overset{(*)}{=} Z^T D Z \\ &\sim \sum_{i=1}^{k} d_i \chi_i^2 \end{aligned} \tag{19}$$

In the above equation series, $Z = RX \sim \mathcal{N}(0, \mathrm{id})$ by rotational invariance of the standard multivariate normal distribution. The calculation of the non-zero coefficients d_i can be accelerated substantially. Let $V = (v_1, ..., v_k) \in \mathbb{R}^{n \times k}$. Note that $\sum_{i=1}^{k} v_i v_i^T = V V^T$, because

$$(VV^T)_{ab} = (v_1(a), ..., v_k(a))(v_1(b), ..., v_k(b))^T = \sum_{i=1}^{k} v_i(a) v_i(b) = \sum_{i=1}^{k} (v_i v_i^T)_{ab} = (\sum_{i=1}^{k} v_i v_i^T)_{ab} \tag{20}$$

Hence

$$L \sum_{i=1}^{k} (v_i v_i^T) L = L V V^T L^T = (LV)(LV)^T \tag{21}$$

Note that $(LV)(LV)^T$ and $(LV)^T(LV) = V^T L^2 V$ have the same non-zero eigenvalues, with the same multiplicity. The latter matrix is merely a matrix of dimension $k \times k$ (k the number of relevant SpaCs), while the former is a $n \times n$ matrix (n the total number of spots on all slides). Further, note that for multiple slides, $L = \mathrm{diag}(\alpha_1 L_1, ..., \alpha_t L_t)$, the calculation of AA^T reduces to $VL(VL)^T = \sum_{j=1}^{t} \alpha_j (V_j L_j)(V_j L_j)^T$, where $V_j \in \mathbb{R}^{n_j \times k}$ is the j-th submatrix of V corresponding to the values on slide j.

To compute p-values, we evaluate the right tail of the null distribution in Equation (19) using the method of Davies [3] and fall back to the slower yet more stable method of Lui [2] in case of non-convergence.

In our application, we choose two disjoint subsets $A \biguplus B \subseteq S$. Starting with some data matrix $X \in \mathbb{R}^{G \times S}$, let $X_A = (x_{a,s})_{a \in A, s \in S} \in \mathbb{R}^{A \times S}$, and let X_B be defined similarly. Then, we perform SPACO for X_A in the spot space $\mathbb{R}^A$ with some suitable scalar product. We obtain k relevant SpaCs (metagenes) $u_1, ..., u_k \in \mathbb{R}^G$. These metagenes induce a pattern space in $\mathbb{R}^B$,$V = \langle X_B u_i; i = 1, ..., k\rangle$. The space V is then used to construct our test statistic.

S8 SPACO Algorithm Pseudocode

Algorithm 1 SPACO projection

Input: samples $\times$ features matrix $X \in \mathbb{R}^{n \times p}$, adjacency matrix $W \in \mathbb{R}^{n \times n}$
Output: A projection $P_{\text{SPACO}} : \mathbb{R}^n \to \mathbb{R}^k$ for some $k \leq n$.

Preprocessing
Remove totally uninformative (constant) features from X.
Center and scale the columns of X to mean 0 and variance 1.

PCA whitening
Perform eigenvalue decomposition of $X^T X = K^T D K \in \mathbb{R}^{p \times p}$ with orthogonal matrix $K = (k_1, \ldots, k_p)$, $k_i \in \mathbb{R}^p$, and $D = \text{diag}(d_1, \ldots, d_p)$, $d_1 \geq d_2 \geq \ldots \geq d_p \geq 0$.
Choose r minimal such that $\sum_{i=1}^{r} d_i \geq c \cdot \sum_{i=1}^{p} d_i$ for some threshold c ($c = 0.95$ by default).
$K_r \leftarrow (k_1, \ldots, k_r) \in \mathbb{R}^{p \times r}$
$D_r \leftarrow \text{diag}(d_1, \ldots, d_r) \in \mathbb{R}^{r \times r}$
$Y \leftarrow X K_r D_r^{-1/2} \in \mathbb{R}^{n \times r}$

Spectral filtering
$L \leftarrow \frac{1}{n} E + \frac{1}{|W|} W \in \mathbb{R}^{n \times n}$
$M \leftarrow Y^T L Y \in \mathbb{R}^{r \times r}$
Perform eigenvalue decomposition $M = U \Lambda U^T$ with orthogonal matrix $U = (u_1, \ldots, u_r)$, $u_i \in \mathbb{R}^r$, and $\Lambda = \text{diag}(\lambda_1, \ldots, \lambda_r)$, $\lambda_1 \geq \lambda_2 \geq \ldots \geq \lambda_r \geq 0$.
Choose k such that $\lambda_k \geq \lambda_{\text{cut}} > \lambda_{k+1}$, where λ_{cut} is determined by resampling.

SPACO projection
$V_k \leftarrow Y(u_1/\sqrt{\lambda_1}, ..., u_k/\sqrt{\lambda_k}) \in \mathbb{R}^{n \times k}$
For a pattern $x \in \mathbb{R}^n$ with mean 0 and variance 1, define the projection P_{SPACO} as

$$P_{\text{SPACO}} : x \mapsto V_k V_k^T L x \in \mathbb{R}^k$$

Algorithm 2 SPACO test

Input: The matrices V_k and L used to construct the SPACO projection, a pattern $x \in \mathbb{R}^n$ with mean 0 and variance 1
Output: The test statistic and a p-value of the SPACO-test

Test statistic
Calculate the non-zero eigenvalues $\sigma_1, ..., \sigma_s$ of $V_k^T L^2 V_k$.
The test statistic is

$$t \leftarrow \|P_{\text{SPACO}}(x)\|\|_L^2 = \left\|V_k^T Lx\right\|_2^2$$

SPACO p-value
Let $q()$ the quantile function of the random variable $T \sim \sum_{i=1}^{s} \sigma_i^2 \chi_i^2$, with i.i.d. chisquared variables $\chi_i^2 \sim \chi^2(1)$.
Calculate the p-value

$$p \leftarrow q(t)$$

S9 Simulation of expression patterns with given Moran's I

Let $\frac{1}{|W|}W = V^T \Lambda V$ the eigenvalue decomposition the symmetric matrix which is used to define Moran's I for a given neighbourhood weight matrix W, with V a unitary matrix $V = (w_1 \dots w_n), w_j \in \mathbb{R}^n$, and diagonal matrix $\Lambda = \mathrm{diag}(\lambda_1, \dots, \lambda_n), \lambda_1 \geq \lambda_2 \geq \dots \geq \lambda_n$. We want to construct an expression pattern $x \in \mathbb{R}^n \backslash \{0\}$,

$$x = \sum_{i=1}^{n} c_i w_i \quad , \; c_i \in \mathbb{R} \tag{22}$$

such that its Moran's I statistic $I(x)$ equals some given value $M \in [-1, 1]$,

$$M = I(x) = \frac{1}{|W|} \frac{x^T W x}{x^T x} = \frac{\sum_{i=1}^{n} \lambda_i c_i^2}{\sum_{i=1}^{n} c_i^2} \tag{23}$$

Multiplying by the denominator and sorting terms, we obtain

$$\sum_{i=1}^{n} (M - \lambda_i) c_i^2 = 0 \tag{24}$$

As $c_i \neq 0$ for at least one i, it follows that this equation only has a solution if $M \in [\lambda_n, \lambda_1]$, and for ease of presentation, we require that $M \in (\lambda_n, \lambda_1)$. For typical neighborhood grids, λ_n will be close to -1 and λ_1 will be close to 1, so this is no serious restriction. A straightforward way to construct suitable coefficients c_i is to sample $s_i \underset{\text{iid}}{\sim} \mathcal{N}(0, 1)$, $i = 1, \dots, n$. Then, define

$$\begin{aligned} s_+ &= \sum_{i, M - \lambda_i > 0} (M - \lambda_i) d_i^2 \\ s_- &= \sum_{i, M - \lambda_i \leq 0} (M - \lambda_i) d_i^2) \end{aligned} \tag{25}$$

and verify by elementary calculations that letting

$$c_i = \begin{cases} d_i / \sqrt{s_+} & \text{if } M - \lambda_i > 0 \\ d_i / \sqrt{s_-} & \text{if } M - \lambda_i \leq 0 \end{cases}, i = 1, \dots, n \tag{26}$$

yields $I(x) = M$ as desired. We finally shift x to mean zero and scale it to unit variance for better visualization. In the simulation for Main Figure 1D, we chose a quadratic 50×50 grid with each point having exactly four neighbours (top, bottom, left, right). Additionally, the leftmost spots shown in the pattern connect to the rightmost spots, and the top spots are neighbors to the bottom spots. In other words, the spots form a torus. On this grid, Moran's I is invariant under horizontal and vertical shifts of the grid and therefore allows us to generate more diverse patterns by a random translocation.

S10 Spot permutation/resampling of features can create spurious anticorrelation with coverage pattern

We observed that the coverage pattern (i.e., the library size per spot, the total read counts mapped to each spot) typically shows a strong spatial structure (Supplemental Figure S3A). As different cell types are likely to contain different amounts of RNA, this is to be expected for most tissues, leading to cell-type specific, spatial variation of library size per spot. Consequently, expression patterns of spatially variable genes often correlate or anticorrelate with this coverage pattern. This can lead to severe artefacts when simulating count numbers of random genes by shuffling/resampling count numbers from an observed gene pattern:

Let $N = (N_i) \in \mathbb{R}^n$ the total coverage count pattern, i.e., N_i is the total number of mapped reads in spot i. In all biological applications known to us, N has a pronounced spatial pattern (Supplemental Figure S3A). Let us consider a gene $x = (x_i) \in \mathbb{R}^n$ and a permuted version of it, $y = (x_{\pi i})$, where π is a random permutation of the spots. Let $\tilde{y}$ the corresponding relative abundance vector of y,

$$\tilde{y}_i = y_i \cdot \frac{1}{N_i} \tag{27}$$

Note that y is a vector with positive entries which fluctuate around some positive mean value. Due to the permuation π, y is uncorrelated to N. Following Equation (27), $\tilde{y}$ will have a tendency to be correlated to $1/N$ and hence anticorrelated to N. Consequently, $\tilde{y}$ will show a non-random spatial pattern (Supplemental Figure S3D). This artefact is dangerous because patterns without spatial structure unintentionally inherit spatial structure from the total coverage pattern N after permutation. We note that this artefact could be the reason why some methods find an excessive number of genes to have a significant spatial pattern. Those methods do not adequately correct for the spatial structure of the count pattern as a confounding factor.

S11 Coverage-adjusted local resampling

For sensitivity and specificity assessment of our method, as well as for checking its robustness against model violations, we need to generate realistic spatial sequencing data with tuneable degrees of non-spatial noise while avoiding the resampling artefacts described above. We developed a coverage-adjusted local resampling method. The basic idea is to take an initial pattern and resample, for each spot, its expression value from the set of its k nearest neighbours. Here, k can be chosen from $k = 0$ (no changes), $k = 1$ (sampling from nearest neighbours) up to $k = \infty$ (random resampling from all available spots). According to the previous section, a naive implementation would lead to resampling artefacts, namely spatially structured patterns that would anticorrelate with the total count pattern. We have therefore developed a method that works with relative frequencies instead of counts, while preserving the total coverage pattern as much as possible.

With the notation from above, let (λ_s^g) the matrix of relative abundances, $\lambda_s^g = \frac{c_s^g}{N_s}$, with c_s^g the count number of feature g in spot s Pick a gene/feature $x$with relative abundance pattern $\lambda^x = (\lambda_s^x)$. Let $\lambda^y = (\lambda_s^y)_{s=1,...,n}$ a "local" bootstrap sample of $\lambda^x = (\lambda_s^x)$. This means that for each spot s, the value λ_s^y is sampled uniformly at random from $\{\lambda_t^x; t \in N(s)\}$, where $N(s)$ is a specified neighbourhood of s, e.g., a circle of fixed radius around s (Supplemental Figure S2A, B). Specifically, for $N(s) = \{1, ..., n\}$, we perform an unrestricted bootstrap which corresponds to erasing all spatial information potentially contained in λ^x. We want to add a perturbed twin y of x to the dataset. This is achieved by letting the relative abundances be

$$\mu_s^g = \frac{\lambda_s^g}{1 + \lambda_s^y}, g \in \{1, ..., p\} \cup \{y\} \tag{28}$$

and the library sizes per spot

$$M_s = (1 + \lambda_s^y) \cdot N_s \tag{29}$$

This leads to the updated count matrix

$$b_s^g = \mu_s^g M_s = \begin{cases} \frac{\lambda_s^y}{1+\lambda_s^y} N_s & g = y \\ c_s^g & g \neq y \end{cases} \tag{30}$$

Note that b_s^y does not need to be an integer. As some methods require integer counts as input, and to avoid bias introduced by rounding of low abundance expression levels, we replace b_s^y with $\lfloor b_s^y \rfloor + \epsilon_s^y, where \lfloor b_s^y \rfloor$ is the largest integer not greater than b_s^y, and $\epsilon_s^y \sim$ Bernoulli(b_s^y mod 1) is a Bernoulli random variable that takes the value 1 with probability equal to the modulus of b_s^y.

S12 Definition of the evaluation metrics

Given gold standard binary labels and a predicted binary labels for a set of items, the **sensitivity** measures the fraction of the actual positive cases that are correctly identified,

$$\text{sensitivity} = \frac{\text{True Positives}}{\text{True Positives} + \text{False Negatives}}$$

The **specificity** measures the fraction of the actual negatives that are correctly identified,

$$\text{specificity} = \frac{\text{True Negatives}}{\text{True Negatives} + \text{False Positives}}$$

The **Receiver Operating Curve (ROC)** is constructed from a set of items carrying a true binary label and a continuous score. Any threshold t splits the items in to classes, which can be interpreted as a predictor, whose sensitivity $\text{sens}(t)$ and specificity $\text{spec}(t)$ is known. The ROC curve is the plot of all points $(1 - \text{spec}(t), \text{sens}(t))$ for all possible thresholds $t \in (-\infty, \infty)$.

The **Adjusted Rand Index (ARI)** measures the agreement between two clusterings of the same set of items, while correcting for the agreement expected by chance. Given two partitions/clusterings U and Von a set of items, let $N = (n_{ij})$ the contingency table formed by these two partitions, i.e., n_{ij} is the number of items in the intersection of cluster i of U and cluster j of V. Let the row sums $a_i = \sum_j n_{ij}$, $i \in U$, the marginal frequencies of the items in cluster i. Similarly, let the column sums $b_j = \sum_i n_{ij}$, $j \in V$, the marginal frequencies of the items in cluster j. The Adjusted Rand Index is defined as

$$\text{ARI} = \frac{\sum_{i,j} \binom{n_{ij}}{2} - A}{\frac{1}{2}\left(\sum_i \binom{a_i}{2} + \sum_j \binom{b_j}{2}\right) - A}$$

where $A = \binom{n}{2}^{-1} \left(\sum_i \binom{a_i}{2}\right) \left(\sum_j \binom{b_j}{2}\right)$.

Normalized Mutual Information (NMI) measures the mutual dependence between two clusterings U and V by comparing the mutual information normalized by their entropies. Specifically, let $N = \sum_{ij} n_{ij}$ and define the marginal probability mass functions $p_U = (a_i/N)_{i=1,\ldots,n}$, $p_V = (b_j/N)_{j=1,\ldots,n}$,and the joint probability mass function $p_{UV} = (n_{ij}/N)_{i,j=1,\ldots,n}$. Then

$$\text{NMI}(U, V) = 2 - 2\frac{H(p_{UV})}{H(p_U) + H(p_V)}$$

References

[1] Myles Hollander, Douglas A Wolfe, and Eric Chicken. *Nonparametric statistical methods*. John Wiley & Sons, 2013.

[2] Huan Liu, Yongqiang Tang, and Hao Helen Zhang. A new chi-square approximation to the distribution of non-negative definite quadratic forms in non-central normal variables. *Computational Statistics & Data Analysis*, 53(4):853–856, 2009.

[3] PG Moschopoulos and WB Canada. The distribution function of a linear combination of chi-squares. *Computers & mathematics with applications*, 10(4-5):383–386, 1984.
