# Supplementary material for: A spectral dimension reduction technique that improves pattern detection in multivariate spatial data
Source: Bioinformatics. 2026 Jan 31;42(2):btag052. doi: 10.1093/bioinformatics/btag052 (PMC12925250; doi:10.1093/bioinformatics/btag052)
Supplement: btag052_Supplementary_Data [file btag052_supplementary_data.zip › SPACO_Supplemental_Figures.pdf]

Supplemental Materials

A spectral Dimension Reduction technique that improves Pattern Detection in multivariate spatial Data

David Köhler, Niklas Kleinenkuhnen, Kiarash Rastegar, Till Baar,
Chrysa Nikopoulou, Vangelis Kondylis, Vlada Michevskaya,
Matthias Schmid, Peter Tessarz, Achim Tresch

Supplemental Figure S1

Split grid validation scheme

A

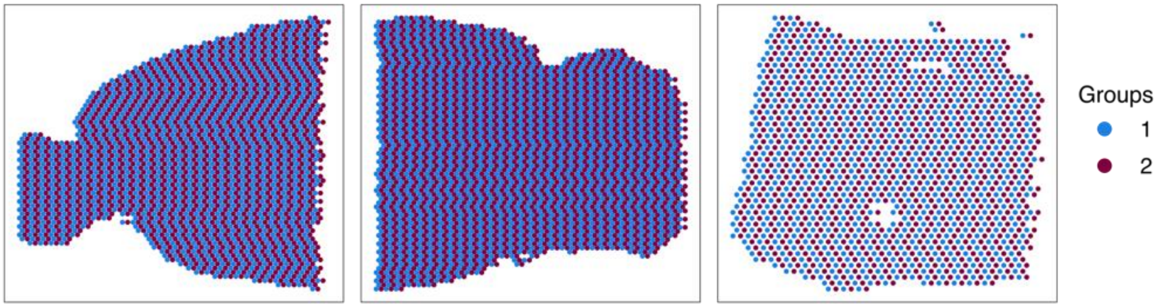

B

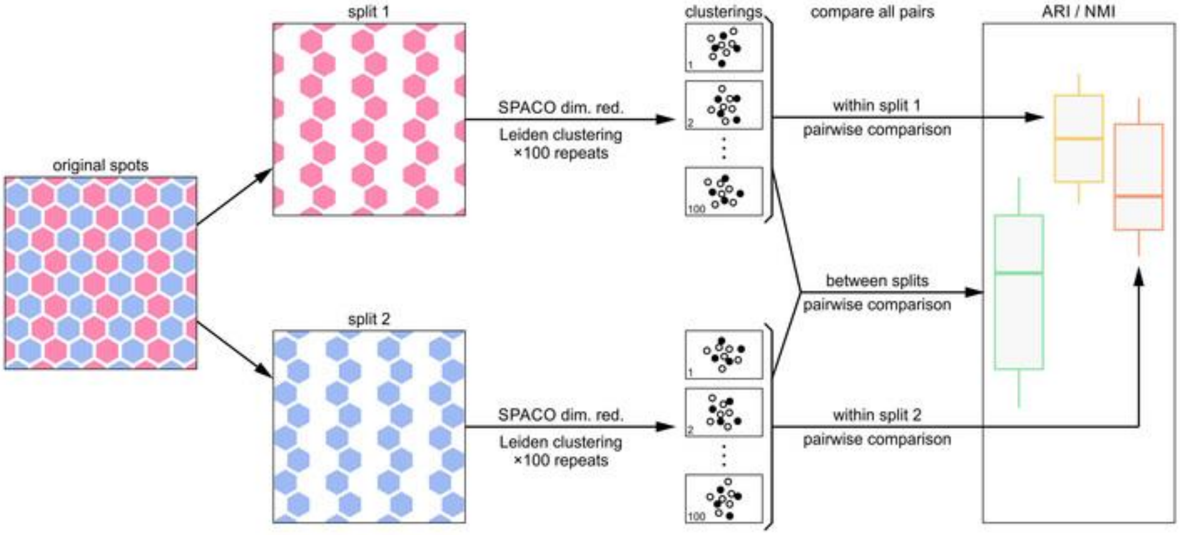

A) Spot grid of the murine anterior & posterior brain (left, middle) and the liver dataset (right) used for clustering consistency evaluation. Red respectively blue spots represent the Split 1 respectively Split 2 dataset.

B) From left to right: The spots on a slide are split into pairs of mutually adjacent spots (red, blue) at the closest horizontal distance. Some spots at the borders may not be paired and are excluded from the analysis. The data set is split into the set of blue and red spots, which are then processed independently by SPACO. The dimension-reduced representation of each dataset is then subjected to Leiden clustering, which is repeated R=100 times to account for the inherent stochasticity of the Leiden method. The similarity of two clusterings is measured by the Adjusted Rand Index (ARI) and the Normalized Mutual Information (NMI). The comparison of clusterings from the same split (yellow, orange) reflects the variability of the Leiden algorithm alone and

therefore represents an upper bound of the achievable ARI/NMD in this dataset. The comparison of the clusterings between Split 1 and Split 2 additionally captures biological variation between neighbouring spots (spatial variation that is at the resolution limit of the respective technology) and measurement noise. The closer the ARI/NMD values are to the upper bound, the more robust the algorithm is to these additional sources of variation.

Supplemental Figure S2

Coverage-adjusted local bootstrapping and denoising

A

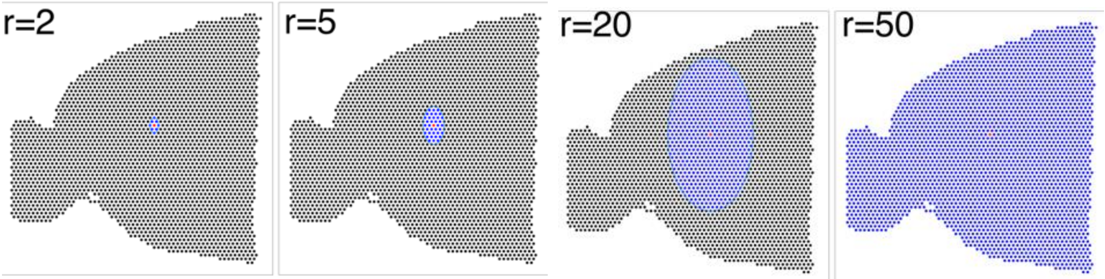

B

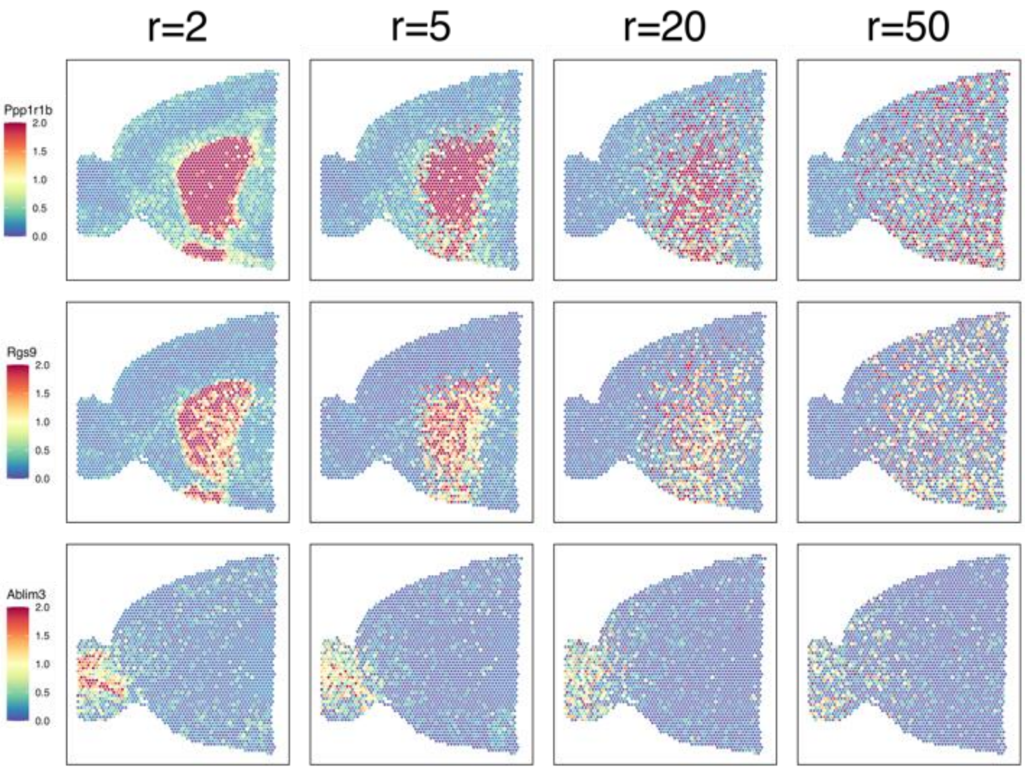

C

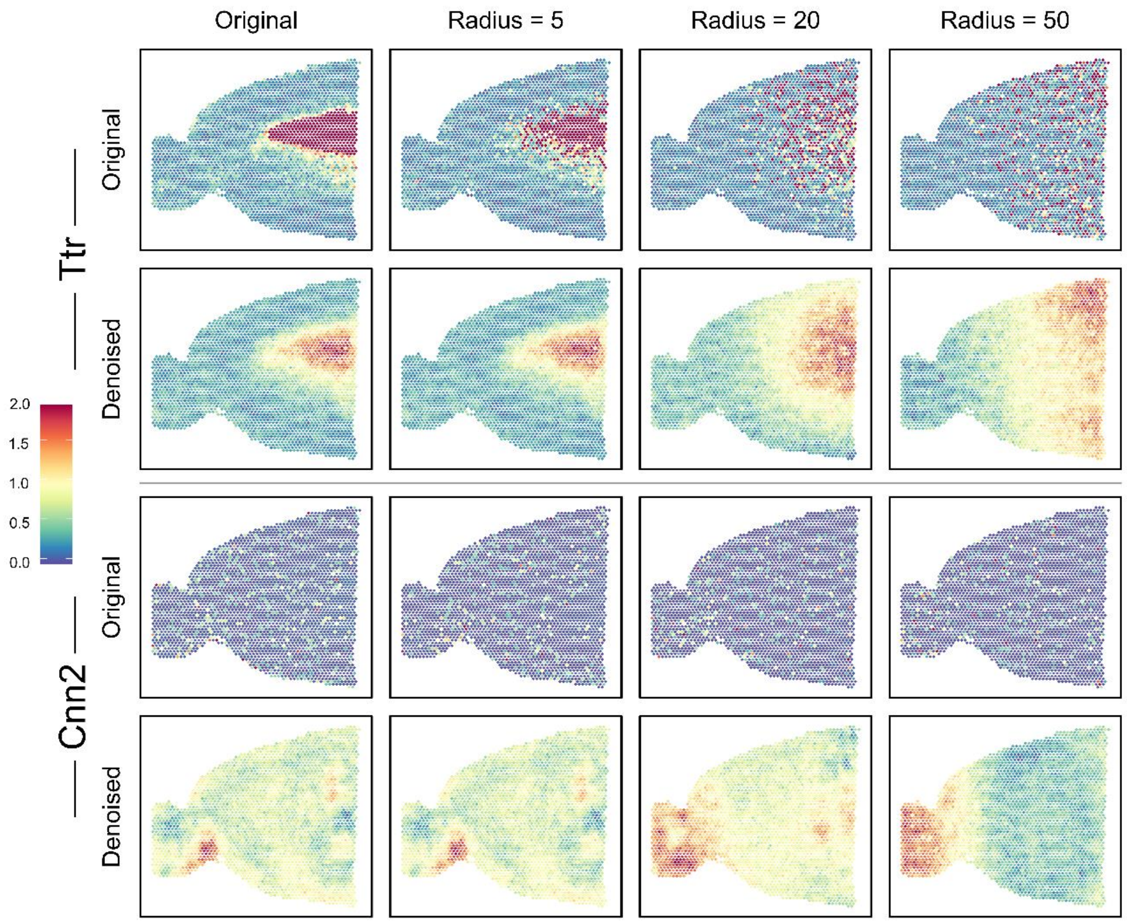

A) Illustration of the neighbourhood (blue spots) of a given spot (red spot) from which values are randomly drawn by local bootstrapping, for different bootstrap radii (r=2, 5, 20, 50) in the mouse forebrain data set.

B) Normalized gene expression of three representative genes (Ppp1r1b, Rgs9 and Ablim3) over increasing noise levels. The neighbourhood radius from which the expression values were resampled is indicated on top of each column.

C) Denoising of spatial patterns (derived from the Ttr gene, first row) and non-spatial patterns (derved from the Cnn2 gene, third row). Columns correspond to different radii of local resampling applied to the original measurements. The second and fourth row show the SPACO projections of the patterns located directly above.

Supplemental Figure S3

Per spot coverage shows a strong spatial pattern that aligns with biological structures, but it can also act as a confounder

The spot coverages of the mouse samples used in this paper show spatial patterns that clearly correspond to known anatomical structures of the mouse brain (Figure S3A). Therefore, as expected, known spatial marker genes of the mouse brain tend to correlate (if expressed in regions of high coverage) or anticorrelate (if expressed in regions of low coverage) with spot coverage. Naïve spot permutation leads to an artefactual anticorrelation of non-spatial genes such as Itgb8 (Figure S3B) and the coverage pattern (Figure S3C). These permutations thus have spatial expression patterns similar to the coverage and a higher level of Moran's I (Figure S3D). Test methods that rely on spot permutation without coverage adjustment may therefore result in an increased false discovery rate of non-spatial genes.

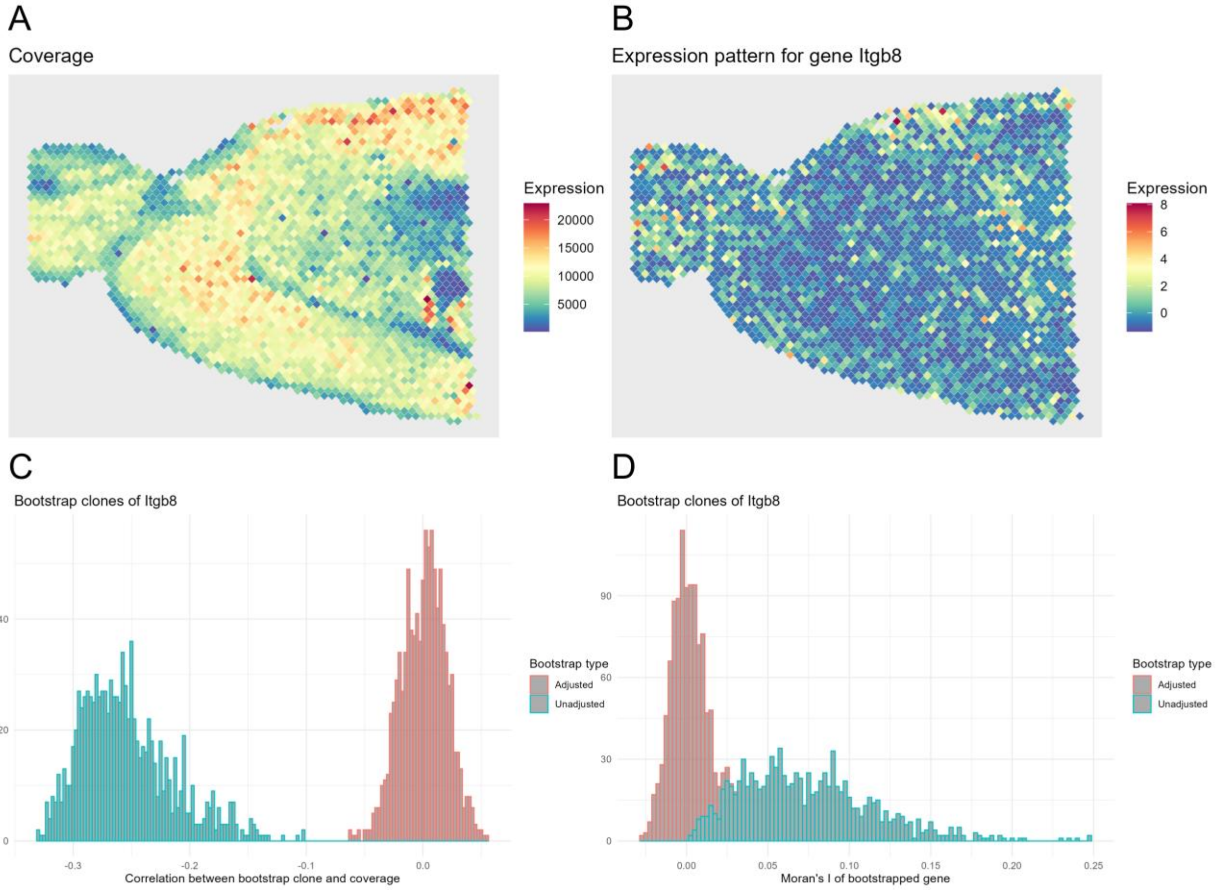

A) Spot coverage pattern in the mouse brain sample.
B) Count pattern of the Itbb8 gene on the same slide as in A).
C) Distribution of the Pearson correlation values of 10,000 bootstrap samples of Itbb8 with the spot coverage pattern in A). Green: Unadjusted bootstrap, red: coverage-adjusted bootstrap.
D) Distribution of Moran's I of 10000 bootstrap samples of Itbg8. Green: Unadjusted bootstrap, red: coverage adjusted bootstrap.

Supplemental Figure S4

GO enrichment analysis of spatially expressed genes

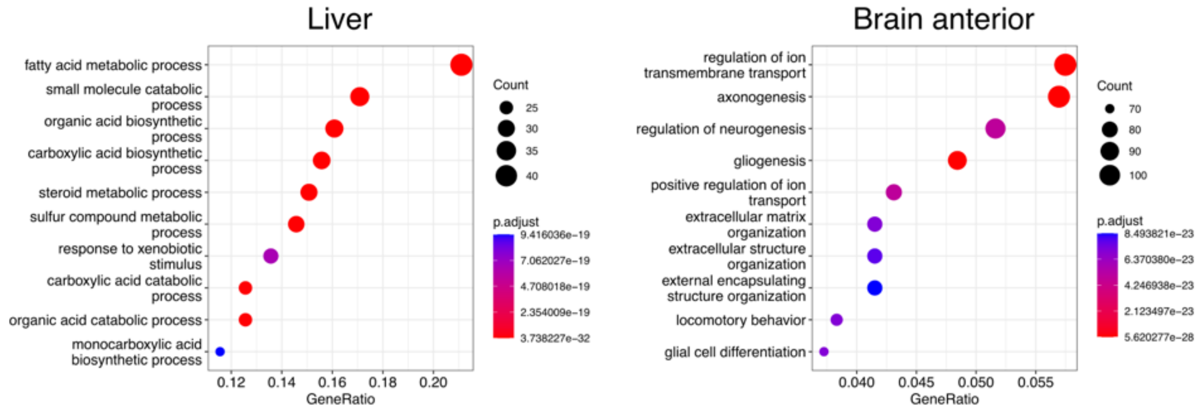

Gene Ontology Analysis of spatially variable genes determined with SPACO that are not listed in the literature as spatially regulated for the murine liver and the anterior brain dataset. Dot size represents the number of counts, adjusted p-value is encoded by colour, and the Gene Ratio on the x-axis denotes the fraction of gene in the respective category which is in the set of investigated genes.
